# Thalamic pathology in frontotemporal dementia: Predilection for specific nuclei, phenotype‐specific signatures, clinical correlates, and practical relevance

**DOI:** 10.1002/brb3.2881

**Published:** 2023-01-07

**Authors:** Mary Clare McKenna, Jasmin Lope, Peter Bede, Ee Ling Tan

**Affiliations:** ^1^ Computational Neuroimaging Group Trinity Biomedical Sciences Institute, Trinity College Dublin Dublin Ireland; ^2^ Department of Neurology St James's Hospital Dublin Ireland

**Keywords:** frontotemporal dementia, MRI, neuroimaging, thalamus

## Abstract

**Background:**

Frontotemporal dementia (FTD) phenotypes are classically associated with distinctive cortical atrophy patterns and regional hypometabolism. However, the spectrum of cognitive and behavioral manifestations in FTD arises from multisynaptic network dysfunction. The thalamus is a key hub of several corticobasal and corticocortical circuits. The main circuits relayed via the thalamic nuclei include the dorsolateral prefrontal circuit, the anterior cingulate circuit, and the orbitofrontal circuit.

**Methods:**

In this paper, we have reviewed evidence for thalamic pathology in FTD based on radiological and postmortem studies. Original research papers were systematically reviewed for preferential involvement of specific thalamic regions, for phenotype‐associated thalamic disease burden patterns, characteristic longitudinal changes, and genotype‐associated thalamic signatures. Moreover, evidence for presymptomatic thalamic pathology was also reviewed. Identified papers were systematically scrutinized for imaging methods, cohort sizes, clinical profiles, clinicoradiological associations, and main anatomical findings. The findings of individual research papers were amalgamated for consensus observations and their study designs further evaluated for stereotyped shortcomings. Based on the limitations of existing studies and conflicting reports in low‐incidence FTD variants, we sought to outline future research directions and pressing research priorities.

**Results:**

FTD is associated with focal thalamic degeneration. Phenotype‐specific thalamic traits mirror established cortical vulnerability patterns. Thalamic nuclei mediating behavioral and language functions are preferentially involved. Given the compelling evidence for considerable thalamic disease burden early in the course of most FTD subtypes, we also reflect on the practical relevance, diagnostic role, prognostic significance, and monitoring potential of thalamic metrics in FTD.

**Conclusions:**

Cardinal manifestations of FTD phenotypes are likely to stem from thalamocortical circuitry dysfunction and are not exclusively driven by focal cortical changes.

## Graphical abstract

Focal, phenotype‐specific, and genotype‐specific thalamic signatures in FTD mirror regional cortical pathology. The structural degeneration of key hubs within thalamocortical circuits is likely to contribute to selective network dysfunction and determine clinical manifestations Radiological thalamic measures have the potential to distinguish FTD subtypes and discriminate FTD from other neurodegenerative disorders and may be developed into biomarkers with practical utility in clinical applications and pharmaceutical trials.

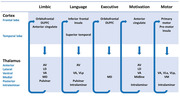

## 1 INTRODUCTION

Frontotemporal dementia (FTD) encompasses a clinically and genetically diverse spectrum of neurodegenerative disorders. While phenotype‐specific cortical signatures and anatomical patterns of hypometabolism are well‐defined, the in‐depth characterization of subcortical pathology is a relatively recent aspiration of quantitative neuroradiology. The contribution of multisynaptic corticothalamic circuits to physiological behavioral, executive, and language functions are relatively well‐established (Bonelli & Cummings, 2007; O'Callaghan et al., 2014). Accordingly, in this review, we first introduce the structural and functional anatomy of the thalamus followed by a systematic review of thalamic involvement across the FTD spectrum stratified according to phenotype, genotype, and pathological subtype.

The thalami are deep paramedian gray matter structures, located superior to the midbrain, joined by the interthalamic adhesion. They are enclosed in a white matter external medullary lamina and separated by a Y‐shaped white matter internal medullary lamina that divides the thalamus into anterior, medial, and lateral anatomical regions. The lateral region is further subdivided into lateral, ventral, and posterior divisions. Each anatomical region contains a subset of thalamic nuclei: anterior thalamic nucleus in the anterior region; medial dorsal and midline nuclei in the medial region; lateral posterior and lateral dorsal nuclei in the lateral division of the lateral region; ventral anterior, ventral lateral, ventral posterolateral, and ventral medial nuclei in the ventral division of the lateral region; and pulvinar, lateral, and medial geniculate nucleus nuclei in the posterior division of the lateral region. The thalamic nuclei also include intralaminar nuclei within the internal medullary lamina and reticular nucleus on the lateral surface of the thalamus (Krauth et al., 2010).

**FIGURE 1:**
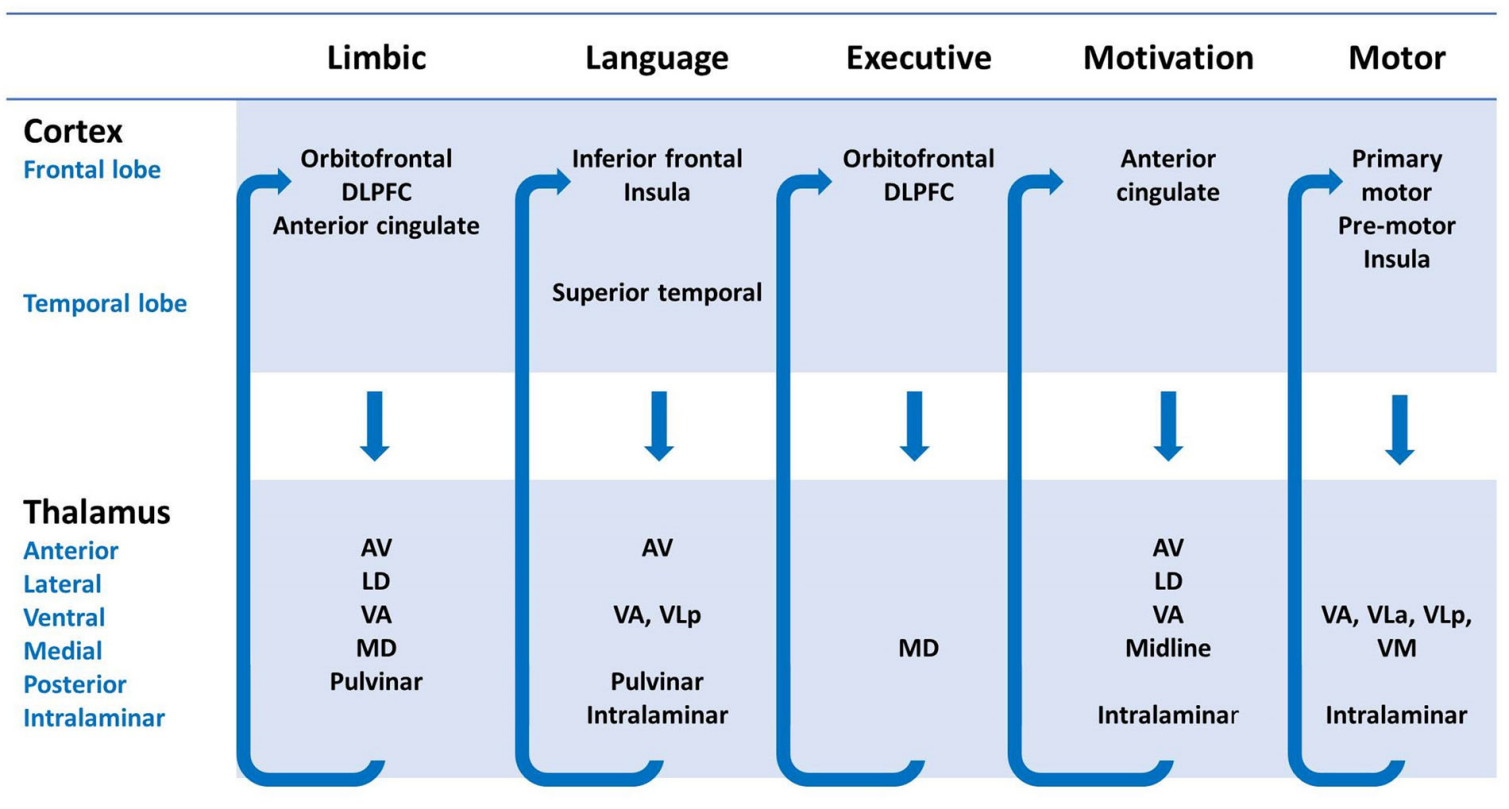
A schematic diagram of distinct thalamocortical circuits, their main thalamic components, cortical projections, and associated physiological role. AV, anterior ventral; DLPFC, dorsolateral prefrontal cortex; LD, lateral dorsal; MD, medial dorsal; VA, ventral anterior; VLa, ventral lateral anterior; VLp, ventral lateral posterior; VM, ventral medial

Functionally, the thalamus mediates a multitude of both sensory and nonsensory processes that extend well beyond these structural boundaries (Figure 1). The sensory functions are classically mapped onto the ventral posterolateral, ventral medial, lateral, and medial geniculate nuclei: specifically, peripheral sensory information (e.g., temperature, pain, vibration, touch, proprioception) is relayed via the ventral posterolateral nuclei, taste and facial sensation via the ventral medial nuclei, visual sensory information via the lateral geniculate nuclei, and auditory sensory information via the medial geniculate nuclei (Schmahmann, 2003). Motor and language functions are relayed by the ventral anterior, ventral lateral, ventral posterolateral, and ventral medial nuclei (Schmahmann, 2003). Limbic processes are conveyed by anterior, ventral anterior, medial dorsal, lateral dorsal, and pulvinar nuclei (Schmahmann, 2003; Vertes et al., 2015). The anterior nuclei give rise to the thalamocingulate tract, an integral part of the Papez circuit that plays a central role in episodic memory (Hornberger et al., 2012; Tan et al., 2014). Associative functions are mediated by midline nuclei: medial dorsal, lateral posterior, and pulvinar nuclei (Schmahmann, 2003; Vertes et al., 2015). This area plays a complex role in cognition and the integration of somatosensory and visuospatial information (Schmahmann, 2003). The intralaminar and reticular nuclei contribute to arousal and alertness (Schmahmann, 2003).

The thalamus is part of a wider network of corticosubcortical circuits including the basal ganglia that mediate cognitive and behavioral functions (Bonelli & Cummings, 2007; O'Callaghan et al., 2014). Each thalamic subregion is linked with specific cortical areas via thalamocortical and corticothalamic projections forming closed‐loop networks (Behrens et al., 2003; Kumar et al., 2017). Macroscopically, the anterior thalamic radiation primarily connects the anterior and medial thalamic regions with the limbic and frontal cortices; the superior thalamic radiation links ventral thalamic regions to the precentral and postcentral gyri; and the posterior thalamic region project to parietal and occipital regions via the posterior thalamic radiation (Zhang et al., 2010). Within these large anatomical labels, there are several specific thalamocortical tracts, such as the thalamocingulate tract connecting the anterior thalamus with the cingulate cortex in Papez circuit (Hornberger et al., 2012; Tan et al., 2014). Functional magnetic resonance imaging (MRI) studies confirm corticothalamic–cortical connections between the prefrontal cortex and mediodorsal and ventral anterior nuclei and anterior thalamic region; the temporal cortex and medial pulvinar and medial geniculate nuclei; the parietal and occipital cortices and lateral pulvinar and lateral geniculate nuclei; the somatosensory cortex with anterior pulvinar and ventral posterolateral nuclei; and the motor and premotor cortex with ventral anterior, ventral lateral, and mediodorsal nuclei (Zhang et al., 2008; Zhang et al., 2010) The disruption of specific thalamocortical circuits has been linked to fairly specific neuropsychological manifestations, such as executive dysfunction, apathy, disinhibition, or depression (Alfano et al., 2022; Bonelli & Cummings, 2007; Brown et al., 2017).

From an imaging perspective, the thalamus is often simplistically considered as a single structure but recent advances in computational imaging have permitted the nuanced appraisal of specific nuclei. With increasing interest in subcortical structures in FTD, we review the existing evidence of thalamic involvement across the FTD spectrum stratified by phenotype, genotype, and pathological subtype. The main objectives of this review are the description of phenotype‐ and genotype‐associated intrathalamic signatures based on consensus research findings, highlighting inconsistencies among published papers, identifying innovative research strategies as well as methodological shortcomings to propose desirable study designs for future initiatives, a synthesis of academic contributions, and reflecting on the potential clinical relevance of thalamic pathology in FTD.

## 2 METHODS

**FIGURE 2:**
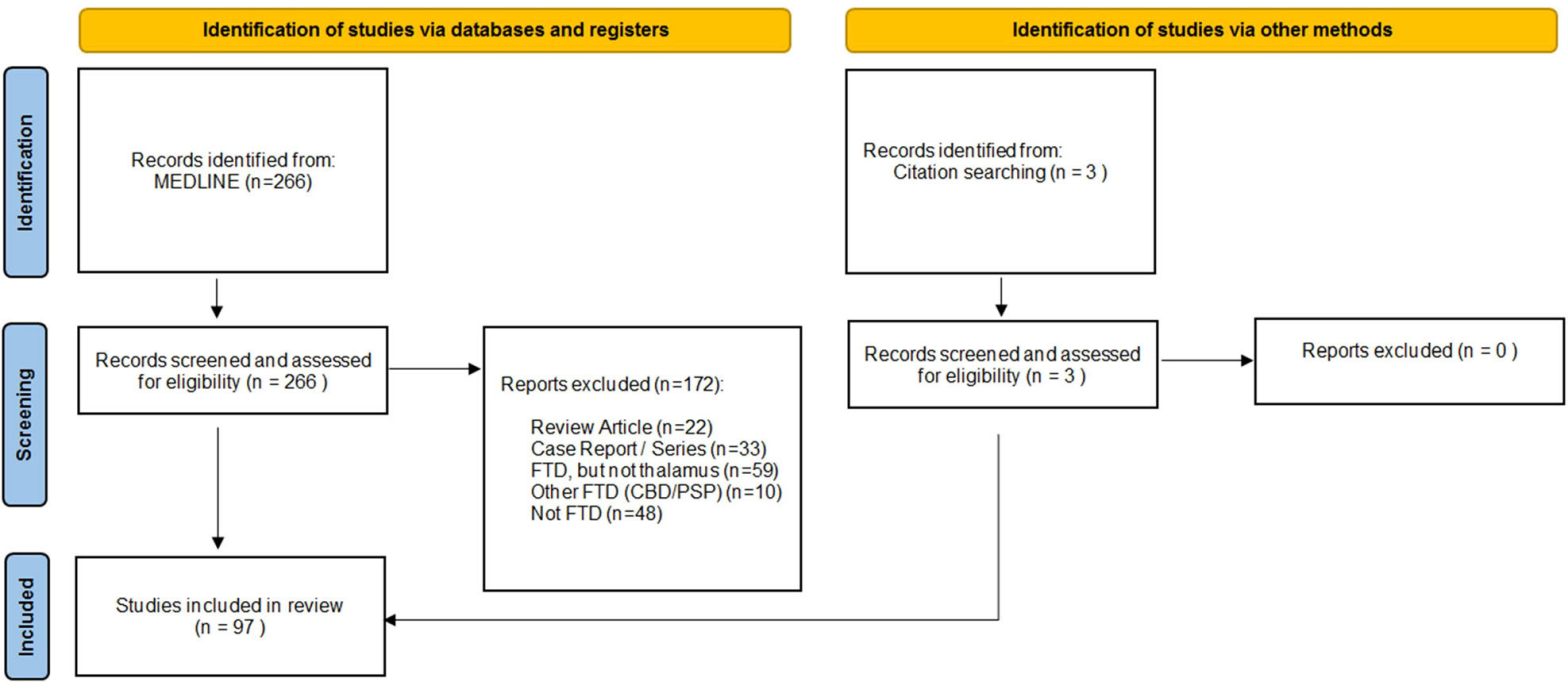
Search strategy, screening, and exclusion of identified papers resulting in the final list of original research papers systematically reviewed as per PRISMA (Preferred Reporting Items for Systematic Reviews and Meta‐analyses) review process. CBD, corticobasal degeneration; FTD, frontotemporal dementia; PSP, progressive supranuclear palsy

A formal literature review was conducted using the PubMed repository (last accessed on May 16, 2022) in accordance with the “preferred reporting items for systematic reviews and meta‐analyses” (PRISMA) guidelines. The following search strategy was used: (“frontotemporal dementia” [Mesh] OR “frontotemporal dementia” [tw] OR “FTD” [tw] OR “frontotemporal lobar degeneration” [tw] OR “FTLD” [tw] OR “C9orf72” [tw] OR “MAPT” [tw] OR “GRN” [tw]) AND (“thalamus”[Mesh] OR “thalam*” [tw] OR “subcortical”) AND (“neuroimaging” [Mesh] OR “MRI” [tw] OR “magnetic resonance imaging” [tw] OR “brain imaging” [tw] OR “neuroimaging” [tw] OR “PET” [tw] OR “positron emission tomography” [tw] OR “pathology” [Mesh] OR “autopsy” [Mesh] OR “neuropathology” [Mesh] OR “post‐mortem” [tw]). The database search was limited to studies written in English that involved human subjects. A single reviewer (MCMcK) individually screened and assessed the 266 records for eligibility. All original research articles that investigated radiological or pathological involvement of the thalamus in FTD were included. Reviews, editorials, and case reports were excluded. Studies limited to corticobasal syndrome and progressive supranuclear palsy (PSP) phenotypes were also excluded. The reference lists of selected articles were reviewed to identify additional, potentially relevant papers (Figure 2). Identified original research articles were individually reviewed for cohort sizes, demographic profile, clinical categorization, genetic information, imaging methods, study design, cross‐sectional versus longitudinal data collection, main findings, anatomical predilection, the battery of accompanying clinical tests, and the presence of presymptomatic or postmortem data.

## 3 RESULTS

A total of 97 original research articles met the inclusion criteria. The majority of these studies were exclusively imaging based (79%; *n* = 77/97), some had both imaging and pathology data (18%; *n* = 18/97), and very few reported pathological data only (3%; *n* = 3/97). The studies were typically unimodal (73%; *n* = 71/97). The most commonly used imaging modality was MRI (88%; *n* = 85/97) including gray matter (77%; *n* = 75/97), white matter (20%; *n* = 19/97), and functional (13%; *n* = 13/97) analyses. A minority of studies used PET imaging (16%; *n* = 16/97). The thalamus was most often considered as a single structure, and seldom segmented into specific nuclei (4%; *n* = 4/97) (Bocchetta et al., 2020; Bocchetta et al., 2021; Chipika et al., 2020; McKenna, Li Hi Shing, et al., 2022). Only a minority of studies were longitudinal (13%; *n* = 13/97) with a mean interval follow‐up of 1.3 ± 0.5 years. The participants were stratified according to phenotype (78%; *n* = 76/97), genotype (46%; *n* = 45/97), or pathology (21%; *n* = 20/97). Presymptomatic familial FTD mutation carriers were occasionally included (19%; *n* = 18/97) (Table 1). The results of these studies are summarized according to phenotype, genotype, and pathological diagnoses (Table S1–S5).

**TABLE 1:** Study characteristics of identified papers

Reviewed studies	*N* = 97
Phenotype	78% (76/97)
bvFTD	63% (48/76)
FTD–ALS	36% (27/76)
svPPA	28% (21/76)
FTLD unspecified	22% (17/76)
nfvPPA	21% (16/76)
PPA unspecified	4% (3/76)
Genotype	46% (45/97)
C9orf72	93% (42/45)
GRN	38% (17/45)
MAPT	33% (15/45)
Other	4% (2/45)
Pathology	21% (20/97)
TDP‐43	70% (14/20)
Tau	40% (8/20)
FUS	25% (5/20)
FTLD unspecified	25% (5/20)
Longitudinal	13% (13/97)
Follow‐up—Average (years)	1.3 ± 0.5 years
Follow‐up—Median (years)	1 ± 0.5 years
Follow up—Range (months)	5–26 months
Presymptomatic	19% (18/97)
Multimodal % (*n*)	27% (26/97)
MRI % (*n*)	88% (88/97)
Gray matter analyses % (*n*)	77% (75/97)
White matter analyses % (*n*)	20% (19/97)
Functional MRI % (*n*)	13% (13/97)
PET % (*n*)	16% (16/97)

*Note*: The table shows a summary of studies evaluating thalamic pathology in FTLD: patient cohorts, study designs, and imaging modalities.

Abbreviations: bvFTD, behavioral variant frontotemporal dementia; C9orf72, chromosome 9 open reading frame 72; FTLD, frontotemporal lobar degeneration; FTD‐ALS, frontotemporal dementia–amyotrophic lateral sclerosis; FUS, fused in sarcoma; GRN, progranulin; MAPT, microtubule‐associated protein tau; MRI, magnetic resonance imaging; nfvPPA, nonfluent variant primary progressive aphasia; PET, positron emission tomography; PPA, primary progressive aphasia; svPPA, semantic variant primary progressive aphasia; TDP‐43, TAR DNA‐binding protein 43.

### 3.1 Phenotypes

The most commonly evaluated clinical phenotypes included behavioral variant FTD (bvFTD) (63%; *n* = 48/76), followed by FTD–amyotrophic lateral sclerosis (ALS) (36%; *n* = 27/76), semantic variant primary progressive aphasia (svPPA) (28%; *n* = 21/76), and nonfluent variant primary progressive aphasia (nfvPPA) (21%; *n* = 16/76). Participants were sometimes grouped together under the umbrella of “unspecified FTD” or “PPA” (Cardenas et al., 2007; Moller et al., 2015; Rombouts et al., 2003) (Table 1). Thalamic atrophy is thought to be most marked in FTD–ALS (McKenna et al., 2021), followed by bvFTD, nfvPPA, and svPPA (Bocchetta et al., 2018; Bocchetta et al., 2020). The degree of thalamic volume loss is sometimes more severe in bvFTD than FTD–ALS (Mann & South, 1993), but differences in symptom duration are seldom accounted for (Li Hi Shing et al., 2021). Postmortem studies have confirmed thalamic atrophy in all FTD phenotypes, sometimes commenting on the affected region (Broe et al., 2003), but seldom mentioning specific nuclei (Broe et al., 2003; Cykowski et al., 2016; Mann & South, 1993; Perry et al., 2017; Rohrer et al., 2010).

#### 3.1.1 Behavioral variant FTD

In bvFTD, diffuse thalamic atrophy (Ahmed et al., 2016, 2019, 2021; Cardenas et al., 2007; Garibotto et al., 2011; Leuzy et al., 2016) involving all thalamic nuclei (McKenna et al., 2022) is often detected. There is particular predisposition to medial dorsal (Bocchetta, Iglesias, et al., 2020; Seeley et al., 2007), lateral dorsal (Bocchetta et al., 2020), and midline (Bocchetta et al., 2020) pathology, which is consistent with postmortem observations (Brettschneider et al., 2014). The pulvinar nuclei (McKenna, Li Hi Shing, et al., 2022; Sturm et al., 2018) may or may not (Bocchetta et al., 2020) be involved. Subtle changes may be captured relatively early, before becoming increasing widespread as the disease progresses (Landin‐Romero et al., 2017; Manera et al., 2019; Seeley et al., 2007). The degree of thalamic atrophy is more prominent in *C9orf72* mutation carriers (Ahmed et al., 2021; Irwin et al., 2013; Sha et al., 2012), which is discussed in more detail below. Morphometric findings are complemented by insights from other imaging modalities such as the reduced integrity of the anterior thalamic radiation (Daianu et al., 2016; Jakabek et al., 2018; Möller, Hafkemeijer, et al., 2015; Spotorno et al., 2020); decreased salience (Ng et al., 2021; Rijpma et al., 2022; Toller et al., 2018; Zhou et al., 2010) and limbic (Farb et al., 2013) network connectivity traversing the thalamic nodes; bilateral thalamic hypometabolism (Diehl‐Schmid et al., 2007; Grimmer et al., 2004; Ishii et al., 1998; Jeong et al., 2005; Leuzy et al., 2016); decreased [(11)C]ABP688) (Leuzy et al., 2016); and increased [18/F]‐THK5351 (Schaeverbeke et al., 2018) radiotracer uptake in the bilateral thalami—the latter indicating nonspecific neurodegeneration (Jang et al., 2018). Paradoxical thalamic hypertrophy, as a potential compensatory mechanism, has also been described in regions projecting to the medial prefrontal cortex (Jakabek et al., 2018).

Thalamic atrophy in bvFTD has been linked to a multitude of cognitive, perceptual, functional, and behavioral impairments. Cognitive impairment is readily associated with anterior thalamic atrophy (Hornberger et al., 2012) that may be preceded by functional working memory network impairment (Rombouts et al., 2003). Detailed neuropsychological testing often reveals impaired object memory (Kumfor et al., 2015), visual memory (Frisch et al., 2013), and design fluency (Possin et al., 2012). Perceptual impairment and psychosis‐like symptoms were also associated with anterior thalamic involvement (Devenney et al., 2021). Functional impairment has been linked to medial dorsal nuclei atrophy (Mioshi et al., 2013). Social cognition and behavioral impairment (Rijpma et al., 2022, 2018, 2017; Toller et al., 2020) may be associated with pulvinar nuclei atrophy. Reduced limbic connectivity of the anterior thalamus has been linked to apathy (Farb et al., 2013). Additionally, there is a trend toward greater posterior thalamic atrophy in those with apathy compared to those without apathy (Links et al., 2009). Thalamic atrophy has also been linked to altered eating behavior (Ahmed et al., 2016) and body composition (Ahmed et al., 2019). Thalamic hypometabolism has been associated with the re‐emergence of primitive reflexes in an admixed group of FTD phenotypes (Matias‐Guiu et al., 2015).

#### 3.1.2 Amyotrophic lateral sclerosis–frontotemporal dementia

Thalamic atrophy (Ahmed et al., 2021; Bede et al., 2018; Machts et al., 2015; Masuda et al., 2016; McKenna et al., 2022) is thought to be particularly striking (McKenna et al., 2021) in ALS–FTD with relatively symmetrical (Bocchetta et al., 2018) involvement of the anterior (anterior ventral), medial (midline, medial dorsal), lateral (lateral dorsal, lateral posterior), ventral (ventral anterior, ventral lateral), and intralaminar nuclei (Bocchetta et al., 2020; Machts et al., 2015; McKenna et al., 2022). There is a particular predilection for the lateral dorsal nuclei (Bocchetta et al., 2020; Machts et al., 2015; McKenna et al., 2022). The posterior (pulvinar, lateral, and medial geniculate) (Chang et al., 2005; McKenna et al., 2022) and additional ventral (ventral medial and ventral posterolateral) (McKenna et al., 2022) aspects are sometimes also implicated. There may be early signs of thalamic atrophy in sporadic ALS with cognitive (Branco et al., 2018) or behavioral (van der Burgh et al., 2020) impairment that does not meet criteria for FTD, but this may not always be the case (Masuda et al., 2016; van der Burgh et al., 2020). Postmortem studies readily confirm widespread thalamic degeneration in ALS–FTD (Mann & South, 1993). Thalamic atrophy may be particularly marked in *C9orf72* hexanucleotide expansion carriers (Agosta et al., 2017; Ahmed et al., 2021; Bede et al., 2018; Irwin et al., 2013; Sha et al., 2012), which is expanded below in detail. While there is a paucity of thalamus‐seeded functional MRI studies in ALS–FTD (Proudfoot et al., 2018), the above findings are supported by white matter analyses that capture reduced superior thalamic radiation integrity (Masuda et al., 2016). Preferentially affected thalamic regions project to motor (Bede et al., 2018; Machts et al., 2015), sensory (Bede et al., 2018; Machts et al., 2015), and limbic (Machts et al., 2015) areas underpinning cognitive correlates (Branco et al., 2018; Machts et al., 2015) and perceptual impairment (Devenney et al., 2021, 2017).

#### 3.1.3 Semantic variant primary progressive aphasia

Thalamic atrophy tends to be relatively subtle in svPPA and may only be a feature of late‐stage disease (Bocchetta et al., 2019). This may explain the strikingly conflicting accounts on the presence (Ahmed et al., 2016) or absence (Bede et al., 2018; Bocchetta, Iglesias Espinosa, et al., 2020; Garibotto et al., 2011) of thalamic involvement in svPPA. If detected, there thought to be a predilection for anterior (anterior ventral), medial (medial dorsal, midline), lateral (lateral dorsal, lateral posterior), or posterior (lateral geniculate) nuclei (Bocchetta et al., 2020; McKenna et al., 2022). Postmortem studies suggest anterior predominant thalamic atrophy (Tan et al., 2014) that occasionally extends to involve the intralaminar (Bocchetta et al., 2020) and more posterior (pulvinar, medial geniculate) nuclei (Mahoney et al., 2011; McKenna et al., 2022). It tends to be left‐lateralized (Ahmed et al., 2016; Bocchetta et al., 2018, 2019; McKenna et al., 2022), yielding the highest asymmetry indexes among FTD phenotypes (Bocchetta et al., 2018). In contrast, morphometric changes may be more pronounced in the right thalamic hemisphere (McKenna et al., 2022). White matter (WM) analyses reveal anterior thalamic radiation degeneration (Downey et al., 2015). In a small FTD cohort that included svPPA, bilateral thalamic hypometabolism was described (Poljansky et al., 2011). Functional analyses show reduced limbic connectivity via the anterior thalamus (Farb et al., 2013). Nuclear imaging studies demonstrate elevated tau‐tracer [^18^F]‐THK5351 binding in the thalamus (Schaeverbeke et al., 2018) indicative of a neurodegenerative process (Jang et al., 2018). Radiological changes in the thalamus have been linked to apathy (Farb et al., 2013), impaired social cognition (Downey et al., 2015), altered eating behavior (Ahmed et al., 2016), as well as auditory symptoms that were specifically associated with medial geniculate nucleus atrophy (Mahoney et al., 2011).

#### 3.1.4 Nonfluent variant primary progressive aphasia

Bilateral (Bede et al., 2018; Garibotto et al., 2011; McKenna, Li Hi Shing, et al., 2022) but left hemisphere predominant thalamic atrophy is typically described in nfvPPA (McKenna et al., 2022; Yoo et al., 2020). Relatively selective anterior (anterior ventral), medial (medial dorsal, midline), lateral (lateral dorsal, lateral posterior), ventral (ventral anterior, ventral lateral, ventral posterolateral, ventral medial), and posterior (medial geniculate) nuclear involvement has been reported (Bocchetta et al., 2020). The pulvinar (McKenna et al., 2022) and sometimes lateral geniculate nuclei (Bocchetta et al., 2020; McKenna et al., 2022) in the posterior region are typically spared. Extensive intrathalamic density reductions are reported (McKenna et al., 2022), particularly in areas projecting to motor regions (Bede et al., 2018). In a small cohort of FTD patients that included nfvPPA, bilateral thalamic hypometabolism was readily captured (Poljansky et al., 2011). Nuclear imaging studies revealed increased tau‐tracer [^18^F]‐THK5351 binding in the thalamus (Schaeverbeke et al., 2018), suggestive of focal neurodegeneration (Jang et al., 2018).

### 3.2 Genotypes

The most common genotypes included in FTD thalamus studies are *C9orf72* (93%; *n* = 42/45), followed by *GRN* (38%; *n* = 17/45) and *MAPT* (33%; *n* = 15/45) mutation carriers as well as less common genotypes such as *TARDBP*, *SOD1*, *FUS*, *TBK2*, or *TREM2* (4%; 2/45). These are low‐incidence disorders, leading to small sample sizes, and often pooled analyses of genetically admixed cohorts are performed. The degree of thalamic atrophy is more marked in familial FTD compared with sporadic FTD (Spinelli et al., 2021), particularly *C9orf72* mutation carriers (Bocchetta et al., 2018, 2021; Bocchetta et al., 2020; Panman et al., 2019). Presymptomatic studies in familial FTD indicate that some of the earliest changes may occur in the thalamus (Cury et al., 2019). Next, we discuss genotype‐specific patterns of thalamic involvement in familial FTD (Bocchetta et al., 2018; Bocchetta et al., 2020).

#### 3.2.1 C9orf72

Thalamic atrophy (Bede et al., 2018; Cajanus et al., 2020; Cash et al., 2018; Lee et al., 2014; Mahoney et al., 2012; McKenna, Li Hi Shing, et al., 2022; Popuri et al., 2018; Sha et al., 2012; Spinelli et al., 2021; van der Burgh et al., 2020) is well‐established in *C9orf72* hexanucleotide expansion carriers, and widely corroborated by pathological studies (Davidson et al., 2016; Irwin et al., 2013; Troakes et al., 2012; Vatsavayai et al., 2016; Yang et al., 2017). It may be symmetrical (Bocchetta et al., 2018), or lateralized. The inconsistency with regard to laterality may stem from small sample sizes, but right‐sided predominance is often observed in *C9orf72*‐associated ALS–FTD (Agosta et al., 2017; Sha et al., 2012), and relative left‐predominance was noted in *C9orf72*‐associated bvFTD (Lee et al., 2014; Sha et al., 2012). The spectrum of thalamic involvement also ranges from relatively focal medial dorsal pathology (Chipika et al., 2020) to more widespread anterior (anterior ventral), lateral (lateral dorsal, lateral posterior), ventral (ventral anterior, ventral lateral), and posterior (pulvinar) thalamic disease burden (McKenna et al., 2022), to encompassing all thalamic nuclei (Bocchetta et al., 2020; Bocchetta et al., 2021). Pulvinar atrophy was previously proposed as a *C9orf72*‐specific trait (Bocchetta et al., 2020; Convery et al., 2020; Lee et al., 2014), but not confirmed by others (Bocchetta et al., 2021; Chipika, Finegan, et al., 2020; Chipika et al., 2020; McKenna et al., 2022). Thalamic atrophy may be too subtle for detection on visual inspection (Sha et al., 2012). In *C9orf72‐*associated ALS–FTD, there may be a preferential involvement of thalamic subregions with motor and sensory thalamocortical projections (Bede et al., 2018). Gray matter findings are complemented by WM analyses that consistently capture anterior thalamic radiation changes in both ALS and ALS–FTD phenotypes (Bede et al., 2013; Floeter et al., 2016; Mahoney et al., 2012). Functional studies invariably detect reduced connectivity in thalamus‐seeded circuits (Shoukry et al., 2020) and the salience network (Lee et al., 2014). ^18^FDG‐PET studies are consistent in identifying bilateral thalamic hypometabolism (Cistaro et al., 2014; Diehl‐Schmid et al., 2019; Soleimani‐Meigooni et al., 2020). The radiological involvement of the thalamus may be associated with elevated serum neurofilament light chains (Cajanus et al., 2020) and cognitive (Floeter et al., 2016; Schönecker et al., 2018), behavioral (Floeter et al., 2016; Lee et al., 2014; Spinelli et al., 2021), and perceptual impairment (Convery et al., 2020; Devenney et al., 2017; Fletcher et al., 2015) in symptomatic disease. In presymptomatic GGGGCC hexanucleotide carriers, similar gray matter (Bertrand et al., 2018, 2021; Cash et al., 2018; Cury et al., 2019; Lee et al., 2016; Panman et al., 2019; Papma et al., 2017; Popuri et al., 2018; Rohrer et al., 2015), white matter (Bertrand et al., 2018; Floeter et al., 2016; Panman et al., 2019; Papma et al., 2017), functional (Lee et al., 2016), and ^18^FDG‐PET (De Vocht et al., 2020; Popuri et al., 2021) thalamus signatures are described as in symptomatic cohorts. Nuclear imaging studies capture presymptomatic synaptic density reduction with a predilection to pulvinar and ventral–posterior thalamic subregions (Malpetti et al., 2021). Presymptomatic metabolic changes in the thalamus may precede structural alterations (Popuri et al., 2021) or changes in cerebrospinal fluid (CSF) markers such as neurofilament light chain (De Vocht et al., 2020). Longitudinal studies suggest that thalamic atrophy remains relatively stable during the presymptomatic phase (Panman et al., 2019), accelerates around phenoconversion (Bocchetta et al., 2021), and either plateaus (van der Burgh et al., 2020) or progresses (Mahoney et al., 2012) thereafter.

#### 3.2.2 GRN

The *GRN* genotype typically involves most thalamic nuclei, particularly the anterior (Spinelli et al., 2021) (anterior ventral [Bocchetta et al., 2021]), medial (medial dorsal and midline [Bocchetta et al., 2021]), and lateral (lateral dorsal [Bocchetta et al., 2021]) regions. There are conflicting reports of posterior (pulvinar [Bocchetta et al., 2020], medial [Bocchetta et al., 2021], and lateral [Bocchetta et al., 2021] geniculate nucleus) and lateral (ventral medial [Bocchetta et al., 2020]) thalamic involvement. This genotype has the highest degree of asymmetric (Bocchetta et al., 2018) thalamic involvement among all genotypes, which may be related to the most commonly associated clinical phenotype, nfvPPA (Bocchetta et al., 2018; Chow et al., 2008). Thalamic atrophy is typically first detected as symptoms emerge (Bocchetta et al., 2021) and seldom evident before this (Feis et al., 2019; Popuri et al., 2018). Presymptomatic studies reveal thalamic hypoperfusion (Dopper et al., 2016) and symmetrical thalamocortical hyperconnectivity involving the salience, language, and default mode networks (Lee et al., 2019). Thalamic involvement in *GRN* has been linked to psychotic symptoms, such as delusions and hallucinations (Sellami et al., 2018).

#### 3.2.3 MAPT

In *MAPT* mutation carriers, widespread thalamic atrophy is typically detected (Bocchetta et al., 2020; Bocchetta et al., 2021), with marked involvement of medial (medial dorsal and midline [Bocchetta et al., 2021]) and lateral (lateral dorsal [Bocchetta et al., 2021]) regions. Reports of posterior thalamic nuclei involvement (pulvinar [Bocchetta et al., 2020] and lateral geniculate [Bocchetta et al., 2021] nuclei) are inconsistent (Bocchetta et al., 2020; Bocchetta et al., 2021). WM analyses reveal loss of the left anterior thalamic radiation integrity compared to controls (Panman et al., 2019).

### 3.3 Histopathology

The most common molecular finding is pTDP‐43 (70%; *n* = 14/20), followed by Tau (40%; *n* = 8/20) and FUS (25%; *n* = 5/20). Pathological diagnoses are sometimes grouped together under the umbrella of FTD/FTLD (25%; *n* = 5/20) (Table 1). Only a minority of FTD studies provide dedicated thalamic histopathology data, either exclusively (3%; *n* = 3/97) or accompanying imaging data (18%; *n* = 17/97). The most marked thalamic involvement is reported in pTDP43‐opathies, followed by Tau‐opathies and then minimal involvement in FUS‐opathies (Bocchetta et al., 2018). Pathology‐specific pattern of thalamic degeneration may be used to differentiate subtypes (Bocchetta et al., 2018). The medial dorsal nucleus is the only nucleus affected in all pathological subgroups (Bocchetta et al., 2020). In addition, there is a significant burden of iron deposition in the thalamus across the FTLD spectrum compared to other neurodegenerative disorders (De Reuck et al., 2014, 2017). Herein, we summarize the thalamic involvement in the pathological subtypes of FTD/FTLD spectrum.

#### 3.3.1 pTDP‐43

The propagation of pTDP‐43 pathology is divided into four sequential stages, with thalamic pathology defining the second pathological stage (Brettschneider et al., 2014). Thalamic atrophy (Cardenas et al., 2007; Pasquini et al., 2020; Yang et al., 2017) is well‐described in pTDP‐43‐opathies, with preferential anterior (Hornberger et al., 2012) and medial (Pasquini et al., 2020) involvement. Thalamic iron deposition is also reported (De Reuck et al., 2017). pTDP‐43 pathology is divided into A, B, and C subtypes that are associated with distinct phenotypes and pathological patterns of thalamic involvement (Bocchetta et al., 2020). Volumetric analyses of pathologically confirmed cases of harmonized classified (Mackenzie et al., 2011) type A pTDP‐43 pathology revealed thalamic atrophy within a group of admixed clinical phenotypes including bvFTD, FTD–ALS, and nfvPPA (Rohrer et al., 2010). This pathological subtype is associated with widespread thalamic atrophy (Harper et al., 2017; Rohrer et al., 2010) implicating thalamic nuclei in the anterior (anterior ventral), medial (medial dorsal, midline, intralaminar), lateral (ventral anterior, ventral lateral, lateral posterior, lateral dorsal), and the posterior (lateral geniculate nucleus) regions (Bocchetta et al., 2020). This contrasts the relatively focal thalamic atrophy observed in type B pTDP‐43 (Bocchetta et al., 2020) that is associated with bvFTD, FTD–ALS, and nfvPPA phenotype (Mackenzie et al., 2011) and the rather limited thalamic involvement noted in pTDP‐43 pathology type C (Bocchetta et al., 2020) that is associated with svPPA or bvFTD phenotype (Mackenzie et al., 2011). In the latter, there may (Bocchetta et al., 2020; Harper et al., 2017; Kawles et al., 2022) or may not (Bocchetta et al., 2020; Kawles et al., 2022) be thalamic involvement at all; if affected, it is limited to the medial dorsal nuclei (Bocchetta et al., 2020). These postmortem observations (Rohrer et al., 2010) have clinical implications as subtle thalamic involvement in type B and C pTDP‐43 pathology may evade radiological detection.

#### 3.3.2 Tau

Thalamic atrophy is commonly observed in Tau‐opathies (Cardenas et al., 2007; Hornberger et al., 2012), further divided into tau‐Pick's, tau‐PSP, tau‐CBD, and FTDP‐17 (Bocchetta et al., 2020). The propagation of tau pathology in Pick's disease is divided into four sequential stages, implicating the thalamus in the second pathological stage (Irwin et al., 2016). The thalamic involvement in Pick's disease (Harper et al., 2017) involves the anterior (anterior ventral), medial (medial dorsal, midline), lateral (lateral posterior, ventral anterior, ventral lateral, ventral posterolateral), and posterior regions (medial geniculate nucleus) (Bocchetta, Iglesias, et al., 2020). There is also thalamic involvement in tau‐PSP (Harper et al., 2017) affecting the medial (medial dorsal, intralaminar) and lateral (ventral anterior and ventral lateral) nuclei; in tau‐CBD (Harper et al., 2017) affecting the anterior (anterior ventral), medial (medial dorsal, midline and intralaminar), and lateral (ventral anterior, ventral lateral, lateral posterior, and particularly lateral dorsal) nuclei; and in FTDP‐17 affecting the medial (medial dorsal, ventral medial, midline), lateral (lateral posterior, ventral lateral, ventral posterolateral), and posterior (medial and lateral geniculate) nuclei (Bocchetta et al., 2020). The different patterns of involvement may be influenced by the associated clinical phenotype (Hornberger et al., 2012).

#### 3.3.3 FUS

The few studies that include FUS‐opathies indicate that there is only minimal thalamic involvement without significant asymmetry (Bocchetta et al., 2018). The medial dorsal nucleus is the only affected thalamic nucleus (Bocchetta et al., 2020). Similar to pTDP‐43‐opathies, iron deposition may also be observed in the thalamus (De Reuck et al., 2017).

## 4 DISCUSSION

There is compelling evidence for thalamic involvement across the clinical, genetic, and molecular spectrum of FTD (Table 2). This is demonstrated by thalamic volume loss involving the anterior, medial, and lateral division nuclei within the lateral region in all clinical phenotypes, genotypes, and most pathological subtypes (Table 2). The consistent involvement of these regions within the corticosubcortical circuits is likely to contribute to some of the cardinal manifestations of FTD such as limbic dysfunction and behavioral and emotional regulation impairment (Bocchetta et al., 2020). There is pan‐thalamic degeneration of most thalamic nuclei in bvFTD and nfvPPA, more selective thalamic involvement in ALS–FTD, and focal thalamic atrophy in svPPA. Thalamic atrophy is more marked in familial FTD. There is diffuse thalamic nuclei atrophy in all genotypes with varying degrees of posterior thalamus involvement. PPA phenotypes and *GRN* genotypes exhibit particularly asymmetric thalamic atrophy. The few available pathology studies demonstrate a variable degree of posterior and ventral thalamic involvement across the pathological subtypes. It is most widespread in the type A subtype of the pTDP‐43‐opathies and tau‐CBD subtypes with only minimal involvement in FUS‐opathies. Thalamic atrophy, among other areas of gray matter degeneration observed in the FTD, may be accompanied by elevated serum (Cajanus et al., 2020; Spotorno et al., 2020) neurofilament light chain, which is a nonspecific marker of neurodegeneration.

**TABLE 2:** A synthesis of focal thalamic volume alterations from published research papers with respect to anatomical predilection

FTLD spectrum	Phenotype				Genotype			Pathological		
Thalamic regions and subregions	bvFTD	FTD–ALS	nfvPPA	svPPA	C9orf72	MAPT	GRN	pTDP‐43	Tau	FUS
Anterior										
Anterior	+	+	+	+	+	+	+	±	±	–
Medial										
Medial dorsal	+	+	+	+	+	+	+	+	+	+
Midline	+	+	+	+	±	+	+	±	±	–
Lateral										
Lateral										
Lateral posterior	+	+	+	+	+	+	+	±	±	–
Lateral dorsal	+	+	+	+	+	+	+	±	±	–
Ventral										
Ventral anterior	+	+	+	–	+	+	+	±	±	–
Ventral lateral	+	+	+	–	+	+	+	+	+	–
Ventral posterolateral	+	±	+	–	±	+	+	–	±	–
Ventral medial	+	±	+	–	±	+	±	–	±	–
Posterior										
Pulvinar	±	±	–	±	+	±	±	–	–	–
Medial geniculate	+	±	+	±	±	+	±	–	±	–
Lateral geniculate	+	±	±	+	±	±	±	+	±	–
Intralaminar	+	+	–	±	±	+	+	±		–

*Note*: The table shows volume reductions in thalamic nuclei across the FTLD spectrum stratified by phenotype, genotype, and pathological subtypes: (+) affected, (±) sometimes affected, and (–) not affected.

Abbreviations: bvFTD, behavioral variant frontotemporal dementia; C9orf72, chromosome 9 open reading frame 72; FTD–ALS, frontotemporal dementia–amyotrophic lateral sclerosis; FUS, fused in sarcoma; GRN, progranulin; MAPT, microtubule‐associated protein tau; nfvPPA, nonfluent variant primary progressive aphasia; svPPA, semantic variant primary progressive aphasia; TDP‐43, TAR DNA‐binding protein 43.

### 4.1 Academic insights

The nuanced characterization of thalamic pathology, either by imaging or histopathological examination, points well beyond descriptive accounts (Table 3). From a conceptual point of view, the ascertainment of focal as opposed to global thalamus degeneration mirroring selective cortical degeneration supports the notion of “what wires together, dies together” (Bak & Chandran, 2012), that is, interconnected brain regions exhibit concomitant neurodegeneration. Conceptually, this is in line with theories of transsynaptic spread of pTDP‐43 (Feiler et al., 2015) and “prion‐like” propagation processes (Nonaka et al., 2013; Smethurst et al., 2016). This also supports observations of co‐occurring deficits in interlinked clinical domains (Grossman et al., 2008). Emerging evidence from presymptomatic studies confirms that pathological change accrues long before symptom onset (Bede et al., 2020; Bertrand et al., 2018; McKenna et al., 2022; Querin et al., 2019; Wen et al., 2019) indicating that neurodevelopmental factors may also be at play (Bede et al., 2020; Lulé et al., 2020). Clustering strategies on large admixed imaging datasets have revealed clinically and radiologically distinct subgroups. For example, various clustering approaches have consistently captured a subcohort of patients with marked frontotemporal change among unselected ALS patients (Bede et al., 2022; Dukic et al., 2022; Tan et al., 2022). Clustering initiatives without a priori hypotheses may successfully uncover pathologically homogenous subgroups that may have distinctive genetic or clinical correlates (Tan et al., 2022). This approach was recently applied to an FTD–ALS cohort that yielded distinct clinical phenotypes with divergent white matter tract involvement (Long et al., 2021).

**TABLE 3:** Key academic insights and clinical relevance of thalamic involvement in the FTD spectrum

Academic Insights	Focal as opposed to global thalamic atrophy
	Phenotype‐ and genotype‐associated thalamic signatures
	Patterns of thalamic involvement mirror regional cortical pathology
	Evidence for “network‐wise” degeneration
	Supports the notion of “prion‐like” propagation in pTDP‐43
	Presymptomatic thalamic changes in mutation carriers
Clinical relevance	Thalamic alterations may precede the radiological detection of cortical change
	Discrimination of phenotypes
	Distinction of FTD from other neurodegenerative conditions such as AD and MCI
	Machine‐learning opportunities
	Putative monitoring role as a biomarker—to be explored
	Predictive value—to be explored
Pragmatic considerations	Fast imaging data acquisition
	Established analysis pipelines
	Semiautomated methods
	Important metrics can be retrieved from T1‐weighted MR data
	Opportunities for reliable single‐voxel spectroscopy
	Putative biomarker role in pharmacological trials—to be explored

### 4.2 Practical relevance

The clinical relevance of thalamic observations stems from the opportunity to capitalize on distinguishing phenotype‐, genotype‐, and pathology‐specific patterns of thalamic involvement in combination with cortical gray matter and white matter neuroimaging signatures. As evidenced by the literature, thalamic involvement can be radiologically detected, and the preferential involvement of specific regions may be computationally characterized. Thalamic signatures may help to distinguish FTD subtypes from controls (Möller et al., 2015), other phenotypes (Bocchetta et al., 2018), genotypes (Bocchetta et al., 2020), pathological subtypes (Bocchetta et al., 2020), and other neurodegenerative disorders such as Alzheimer's disease (Meysami et al., 2022; Möller et al., 2015). There are preliminary indications that using the volume of individual thalamic nuclei, rather than volume of the entire thalamus, may have better discriminating power (Bocchetta et al., 2018; Bocchetta et al., 2020). While the optimal combination of thalamic volumetric measurements is yet to be determined, a single study demonstrated that the volume of the pulvinar nuclei accurately differentiates *C9orf72* from *MAPT* genotypes, and varying combinations of anterior, lateral, medial, and intralaminar nuclei volume reliably discriminate pathological subtypes (Bocchetta et al., 2020). The increasing availability of uniformly acquired normative datasets may help the radiological interpretation of single patients with FTD or suspected FTD (McKenna et al., 2022; McKenna et al., 2022; Tahedl et al., 2021). Machine learning applications are increasingly applied to large FTD and ALS–FTD datasets (McKenna et al., 2022). MRI‐based classification models use discriminatory MRI features to categorize single‐subject MRI data into diagnostic groups. Feature selection in ALS–FTD spectrum disorders typically focuses on cortical gray matter thickness, volumes, and white matter metrics (Bede et al., 2022; Egger et al., 2020; Grollemund et al., 2019; Kim et al., 2019; McKenna, Murad, et al., 2022; Premi et al., 2016; Schuster et al., 2016, 2017) rather than subcortical volumes; this is likely because subcortical volumes are considered as a whole instead of the inclusion of nucleus‐based metrics in the models. Thus, the addition of thalamic nuclei and thalamic radiation integrity metrics may improve the classification accuracy of such models (Bede et al., 2021). Presymptomatic thalamic atrophy observed in *C9orf72* genotype may be used to ascertain and track disease burden prior peridiagnostic biomarker changes, such as CSF neurofilament light chain concentration alterations (De Vocht et al., 2020). From a medical education point of view, the thalamus is continued to be predominantly linked to sensory function. The importance of thalamus‐mediated cognitive, behavioral, and extrapyramidal motor function needs to be emphasized at an undergraduate level and illustrated in a clinical context such as FTD for future generations of physicians. Presymptomatic studies suggest that pathological changes may be detected several years, sometimes decade before symptom onset (Bertrand et al., 2018; Querin et al., 2019). Presymptomatic insights and the observation that widespread pathological changes can be detected by the time diagnostic criteria are met would suggest that the window for effective pharmacological intervention with true disease‐modifying potential may fall into the presymptomatic or prodromal phase of the disease. The recognition of considerable disease burden around the time of diagnosis should hasten recruitment into clinical trials very early in the course of the disease and may ultimately pave the way for presymptomatic clinical trials in mutation carriers (Querin et al., 2022).

### 4.3 Study limitations

Our review also highlights the most common methodological shortcomings of thalamic studies that should be considered in the design of future research initiatives. First, heterogenous groups of different FTD phenotypes, genotypes, and pathological subtypes are sometimes admixed to boost sample sizes, but this precludes the precision characterization of subtype‐specific thalamic signatures. Despite this, sample sizes often remain relatively small, in part because of the rarity of these conditions. Second, most studies consider the volume of the entire thalamus, with only a minority of studies using emergent methods to quantify the volume of individual thalamic nuclei. Third, the majority of imaging studies adopt a single modality approach, overwhelmingly focusing on the thalamic gray matter. Multimodal imaging strategies, integrating structural, functional, metabolic, and connectivity‐based observations are not only more informative but reveal more about the role of thalamic pathology in the context of thalamocortical circuitry dysfunction. Fourth, while several studies ascribe deficits in specific clinical domains to thalamic atrophy, direct clinicoanatomical correlations are somewhat contentious (Verstraete et al., 2015) as cognitive and behavioral functions are mediated by multisynaptic networks with multiple gray and white matter components. Additionally, there is a disproportionate focus on the more common FTD phenotypes and thalamic pathology in low‐incidence entities such as primary lateral sclerosis (PLS)‐associated FTD, complicated hereditary spactic paraplegia (HSP), ALS–FTD, or spinal‐bulbar muscular atrophy (SBMA)‐associated frontotemporal dysfunction is relatively under‐investigated (Chipika et al., 2020; Christidi et al., 2018; Finegan et al., 2019; Finegan et al., 2019; Finegan et al., 2020) despite radiological evidence of frontotemporal pathology in PLS (Bede et al., 2022; Finegan et al., 2021), hereditary spastic paraplegia (Mulkerrin et al., 2022), and to a lesser extent in spinal bulbar muscular atrophy (Pradat et al., 2020; Querin et al., 2018). Anatomically elusive clinical symptoms such as fatigue have been repeatedly linked to thalamic changes (Clark et al., 2018; Li Hi Shing et al., 2019; Seok et al., 2022), but compelling evidence for direct associations is lacking (Li Hi Shing et al., 2021). Executive function, language, motivation, and limbic functions are the main nonsensory functions linked to thalamic nuclei, but thalamic nuclei also mediate social cognition and theory of mind (ToM)‐related functions (Ferguson & Gao, 2018; Wolff & Vann, 2019). ToM deficits are increasingly recognized in a multitude of FTD phenotypes (Burke et al., 2016; Burke, et al., 2016) and the contribution of thalamic pathology should be systematically investigated in these conditions. Pseudobulbar affect (PBA) is a clinical syndrome which is classically linked to corticobulbar disconnection, but more recent models implicate cortical–limbic–subcortical–thalamic–pontocerebellar network dysfunction (Bede & Finegan, 2018; Finegan et al., 2019; King & Reiss, 2013). Finally, the involvement of sensory nuclei is seldom appraised, despite evidence of marked ventral posterolateral and ventromedial thalamic volume loss in GGGGCC hexanucleotide repeat expansion carriers (Chipika et al., 2022) in ALS and ALS–FTD (Chipika et al., 2022). From a sensory perspective, the spinothalamic and dorsal column–medial lemniscus pathways are rarely investigated, even though the integrity of these tracts can now be reliably assessed at both the spinal and cerebral levels (Bede et al., 2012; El Mendili et al., 2019).

### 4.4 Methodological considerations

Thalamic integrity may be evaluated with relative ease and a number of robust open‐source software libraries are available to retrieve a variety of thalamus metrics. The observation that in most FTD subtypes thalamic atrophy is an early feature (McKenna, Lope, et al., 2022) and may precede characteristic cortical atrophy provides a strong rationale for quantitative thalamus imaging in FTD. Total thalamus volume and the volumes of specific nuclei can be estimated from high‐resolution three‐dimensional T1‐weighted data (Iglesias et al., 2018), which are routinely acquired in clinical protocols as part of the diagnostic workup; therefore, there are no additional time or cost implications for acquiring raw data for post hoc thalamic analyses. Similarly, shape deformation analyses also rely on three‐dimensional T1‐weighted images eliminating the need for additional data acquisition and scanning costs (Patenaude et al., 2011). One of the challenges of cortical single‐voxel magentic resonance spectroscopy (MRS) is the consistency in voxel placement (Christidi et al., 2022), which is not a problem in thalamus spectroscopy as the structure is readily identified on localizer scans (Sharma et al., 2011). As the thalami are paired structures, commenting on symmetry or asymmetry based on retrieved integrity indices is very straightforward. Similarly, longitudinal statistical models are not challenging to implement (Chipika et al., 2019; Schuster et al., 2015; Zarkali et al., 2022). While overall thalamic volumes are often evaluated and “overall” thalamic metabolism appraised, the thalamus consists of over 50 cytologically and functionally distinct nuclei (Behrens et al., 2003) with distinguishing cortical projection patterns (Bede et al., 2018), physiological roles (Bosch‐Bouju et al., 2013), developmental origin (Blackshaw et al., 2010), and vascular supply (Schmahmann, 2003). The main caveat of assessing the thalamus as a single structure, either by volumetric (Machts et al., 2015), metabolic (Cistaro et al., 2014), spectroscopic (Sharma et al., 2011), or vertex‐based method (Bede et al., 2013), is potentially averaging imaging metrics across preferentially affected and unaffected regions, thus reducing detection sensitivity for pathological change. A number of innovative computational strategies have been developed and validated, most of which are available as open‐source pipelines, to parcellate the thalamus either by cortical connectivity patterns (Bede, 2017; Behrens et al., 2003; Johansen‐Berg et al., 2005; Tu et al., 2018; Zhang et al., 2017) or based on the histological data (Iglesias et al., 2018). Compared to cortical pipelines, quantitative thalamus imaging remains somewhat overlooked, despite simplicity of implementation, moderate computational time requirement, and the availability of normative datasets.

### 4.5 Future directions

Given the academic and clinical relevance of thalamic measures in FTD, standard clinical imaging protocols should invariably include a high‐resolution three‐dimensional T1‐weighted pulse sequence and basic thalamus metrics should be routinely interrogated. A relatively short diffusion tensor imaging protocol offers ample opportunities for additional white matter analyses to evaluate the integrity of thalamic projections. It seems imperative that multimodal imaging protocols are implemented in the research setting so that the comparative detection sensitivity, prognostic value, and monitoring potential of the various metrics can be contrasted and the best‐performing indices for future clinical use and as biomarkers in future pharmacological trials can be selected. Future academic studies should routinely include disease controls in addition to healthy controls to assess the specificity of thalamic alterations to specific FTD subtype. Cross‐sectional studies of patients with varied symptom duration reveal very little about the dynamic molecular process driving FTD; therefore, carefully designed multi‐timepoint imaging studies are required with uniform follow‐up intervals to establish the natural history of disease burden propagation. As with other neurodegenerative conditions, longitudinal studies should ideally include presymptomatic mutation carriers to clarify the value of radiological metrics in predicting phenoconversion and contribute to academic debates such as neurodevelopmental versus neurodegenerative processes and the existence of compensatory and adaptive mechanisms in neurodegeneration.

## 5 CONCLUSIONS

FTD is associated with phenotype‐, genotype‐, and pathological subtype‐specific thalamic signatures. Thalamic degeneration is likely to contribute to the diverse manifestations observed clinically as a key hub of subcortical–cortical networks. Large, pathologically and biomarker‐supported longitudinal imaging studies are required with a standardized imaging and clinical protocol for the nuanced characterization of thalamic pathology in FTD in order to develop clinically meaningful biomarkers centered on thalamic changes.

## AUTHOR CONTRIBUTIONS

The manuscript was drafted by Mary Clare McKenna and revised for intellectual content by Jasmin Lope, Peter Bede, and Ee Ling Tan.

## CONFLICT OF INTEREST

The authors declare no conflict of interest.

### PEER REVIEW

The peer review history for this article is available at https://publons.com/publon/10.1002/brb3.2881.

## Supporting information

Supplementary Table 1. Neuropathology
Supplementary Table 2. Structural imaging
Supplementary Table 3. White matter imaging
Supplementary Table 4. Functional MRI
Supplementary Table 5. Positron emission tomography.

## Data Availability

Relevant data from original research studies pertaining to thalamic changes are systematically summarized in Tables S1–S5.
