# Supplementary material for: Thalamic pathology in frontotemporal dementia: Predilection for specific nuclei, phenotype‐specific signatures, clinical correlates, and practical relevance
Source: Brain Behav. 2023 Jan 7;13(2):e2881. doi: 10.1002/brb3.2881 (PMC9927864; doi:10.1002/brb3.2881)
Supplement: Supplementary file 1 — Supplementary Table 1. Neuropathology Supplementary Table 2. Structural imaging Supplementary Table 3. White matter imaging Supplementary Table 4. Functional MRI Supplementary Table 5. Positron emission tomography. [file BRB3-13-e2881-s001.docx]

**Supplementary Table 1**. Neuropathology

| **Neuropathology** | | | | | | |
| --- | --- | --- | --- | --- | --- | --- |
| **First author, year of publication** | **Patient groups and cohort sizes** | **Study design** | **Neuropathology** | **Follow-up duration** | **Imaging modality** | **Key outcomes** |
| Brettschneider et al, 2014 (1) | FTLD-TDP n=39  (bvFTD n=39)  (C9orf72 n=12; GRN n=6) | Cross-sectional  Case-series | All cases | N/A | N/A | Four sequential patterns of TDP-43 pathology were described. The second pattern included increasing burden of pathology pTDP-43 in the thalamus. |
| Kawles et al, 2022 (2) | FTLD-TDP type C n=10  (svPPA n=7; bvFTD n=3) | Cross-sectional  Case-series | All cases | N/A | N/A | Thalamus, subthalamus and globus pallidus were only mildly affected or spared from TDP-type C pathology. |
| Yang et al, 2017 (3) | C9orf72 positive bvFTD n=13  Sporadic bvFTD n=8  Sporadic ALS n=7  Controls n=7 | Cross-sectional  Case-control | All cases | N/A | N/A | Thalamus degeneration was only detected in C9orf72 positive bvFTD, and in a similar extent in those with and without psychosis.  There was no significant difference in von Economo neuron density or thalamus degeneration between bvFTD with and without C9orf72 repeat expansion. |

**Supplementary Table 2**. Structural imaging

| **Structural (Segmentation and Volumetry)** | | | | | | |
| --- | --- | --- | --- | --- | --- | --- |
| **First author, year of publication** | **Patient groups and cohort sizes** | **Study design** | **Neuropathology** | **Follow-up duration** | **Imaging modality** | **Key outcomes** |
| Ahmed et al, 2016 (4) | bvFTD n=19  svPPA n=15  AD n=15  Controls n=25 | Cross-sectional | N/A | N/A | MRI -VBM | Increased caloric intake was associated with bilateral thalami, cingulate and cerebellar atrophy in bvFTD; and with left thalamus, nucleus accumbens, cerebellum, orbitofrontal, and cingulate cortical atrophy in svPPA. |
| Ahmed et al, 2019 (5) | bvFTD n=28  AD n=16  Controls n=19 | Cross-sectional | N/A | N/A | MRI - VBM | The changes in body composition in FTD are correlated with GM atrophy involving the autonomic control and reward processing networks including the thalamus |
| Ahmed et al, 2021(6) | bvFTD n=58 (C9orf72 mutation carriers n=17)  ALS-FTD n=41 (C9orf72 mutation carriers n=12)  Controls n=58 | Cross-sectional | N/A | N/A | Cortical volumes  White matter volumes  Subcortical volumes | There was marked subcortical atrophy, involving the thalamus, amygdala, hippocampus and striatum in bvFTD and ALS-FTD compared to ALS and controls. In C9orf72 mutation carriers, there was more prominent thalamic involvement. This loss of volume of the frontoinsular network was associated with abnormal behaviour. |
| Bede et al, 2018 (7) | bvFTD n=10  nfvPPA n=11  svPPA n=5  C9orf72 positive ALS-FTD n=14  C9orf72 negative ALS-FTD n=12  ALS without cognitive impairment n=36  Controls n=50 | Cross-sectional | N/A | N/A | Cortical thickness  Subcortical volume, density and connectivity-based segmentation | In nfvPPA, there was bilateral thalamic atrophy, with density reductions in areas projecting to motor regions.  In C9orf72 positive and negative ALS-FTD, there was bilateral thalamic atrophy with density reductions in sub-regions connected to motor and sensory cortical areas that were more marked in C9orf72 mutation carriers.  There was no difference in thalamic volume in bvFTD, svPPA and in ALS with no cognitive impairment. |
| Bocchetta et al, 2018 (8) | FTD n=341  Phenotypes: (bvFTD n=141; svPPA n=76; nfvPPA n=103; FTD-ALS n=7; PPA unspecified n=14)  Genotypes: (MAPT n=24; C9orf72 n=24; GRN n=15)  Pathology: (Tauopathy n=40; TDP-43opathy n=61; FUSopathy n=3)  Controls n=99 | Cross-sectional  Case control | N=104 (TDP43opathies n=61; Tauopathies n=40; and FUSopathies n=3) | N/A | MRI -Volumetry | All clinical phenotypes, genotypes and pathological subtypes of FTD had smaller thalami than controls, except those with FUSopathies.  The thalamus was most affected in C9orf72 genetically, TDP-43opathies pathologically and FTD-ALS clinically.  The most asymmetric thalamic volumes were detected in unspecified PPA, svPPA and GRN genotypes. |
| Bocchetta et al, 2019 (9) | svPPA n=24  Controls n=72 | Cross-sectional  Case control | N/A | N/A | Cortical volumes  Subcortical volumes with medial temporal region segmentation | In late stage svPPA, there is left thalamus involvement |
| Bocchetta et al, 2020 (10) | FTLD TDP-43 Type C n=19  Controls n=81 | Longitudinal (n=14)  Case control | All cases | Not specified | MRI - Volumetry | This study defined the sequential stages of radiological and pathological involvement in FTLD-TDP type C. There was no radiological involvement of the bilateral thalami. |
| Bocchetta et al, 2020 (11) | FTD n=402  Phenotypes: (bvFTD n=180; svPPA n=85; nfvPPA n=114; FTD-ALS n=8; PPA unspecified n=15)  Genotypes: (MAPT n=27; C9orf72 n=28; GRN n=18)  Pathology: (Tauopathy n=37; TDP-43opathy n=38; FUSopathy n=4)  Controls n=104 | N/A | N=79 (Tauopathy n=37; TDP-43opathy n=38; FUSopathy n=4) | N/A | MRI – Volumetry with thalamic nuclei segmentation | The volume of individual thalamic nuclei provided higher accuracy than whole thalamus volume in discriminating FTD groups.  The mediodorsal nucleus was affected in all FTD groups; and the pulvinar nucleus was only affected in C9orf72. |
| Bocchetta et al, 2021(12) | Pre-symptomatic MAPT n=47  Mild symptomatic MAPT n-13  Symptomatic MAPT n=20  Pre-symptomatic GRN n=125  Mild symptomatic GRN n=30  Symptomatic GRN n=43  Pre-symptomatic C9orf72 n=107  Mild symptomatic C9orf72 n=32  Symptomatic C9orf72 n=63  Controls n=298 | Cross-sectional  Case control | N/A | N/A | Cortical volumes  Subcortical volumes | In pre-symptomatic C9orf72 mutation carriers, there was thalamic atrophy mainly involving the laterodorsal, pulvinar and lateral geniculate nuclei. This became more widespread in symptomatic C9orf72 mutation carriers.  In symptomatic MAPT and GRN mutation carriers, widespread thalamic atrophy was more marked in the mediodorsal, laterodorsal and midline nuclei. The anteroventral region was also affected in GRN carriers. |
| Branco et al, 2018 (13) | ALS n=50  (Cognitively impaired ALS n=12)  Controls n=38 | Cross-sectional  Case control | N/A | N/A | Cortical thickness  Subcortical volume  DTI | Cognitively impaired ALS patients had reduced left thalamus volume compared to non-impaired ALS patients. This correlated with cognitive performance. |
| Cajanus et al, 2020 (14) | FTLD C9orf72 carrier n=26; (bvFTD n=19, PPA n=5; FTD-ALS n=2)  FTLD C9orf72 non-carrier n=52; (bvFTD n=35; PPA n=14; FTD-ALS n=3) | Longitudinal (n=11) | N/A | 23 months (8-42 months) | Cortical thickness  Subcortical volume | Serum neurofilament light chain were higher in C9orf72 repeat expansion mutation carriers versus non-carriers.  High serum neurofilament light change levels were significantly correlated with shorter survival time, frontal cortical atrophy rate and subcortical GM atrophy rate – especially involving thalamus, caudate and pallidum |
| Cardenas et al, 2007 (15) | FTD n=22 (ALS-FTD n=5)  Controls n=22 | Cross-sectional  Case control | N=5 (Pick’s disease n=2; FTD-ubiquitin n=2; ALS-FTD n=1) | N/A | Morphometry | In FTD, there was subcortical and midbrain atrophy involving thalamus, pons, superior and inferior colliculi. |
| Cash et al, 2018 (16) | Pre-symptomatic GRN n=65  Symptomatic GRN n=12  Pre-symptomatic C9orf72 n=40  Symptomatic C9orf72 n=25  Pre-symptomatic MAPT n=23  Symptomatic MAPT n=10  Controls n=144 | Cross-sectional  Case control | N/A | N/A | VBM | In pre-symptomatic and symptomatic C9orf72 mutation carriers, there was bilateral thalamic volume loss. |
| Chang et al, 2005 (17) | ALS without cognitive or behavioural impairment n=10  FTD-ALS n=10  Controls n=22 | Cross-sectional  Case control | N/A | N/A | VBM | In ALS and ALS-FTD, there was left posterior thalamus atrophy in both subgroups, with a trend towards more significant thalamic involvement in ALS-FTD. |
| Chipika et al, 2020 (18) | C9orf72 positive ALS n=12 (ALS-FTD n=8/12)  C9orf72 negative ALS n=88 (ALS-FTD n=7)  PLS n=33  Controls n=117 | Cross-sectional  Case control | N/A | N/A | Total intracranial volumes  Thalamus  Segmentation  Vertex analyses  Morphometry | In combined group of ALS and ALS-FTD stratified by genetics, C9orf72 negative group showed involvement of the motor, sensory, intralaminar, anteroventral, medial geniculate, lateroposterior and mediodorsal nuclei; C9orf72 positive group showed involvement of only mediodorsal nuclei. Vertex analyses showed similar superior, inferior and posterior thalamic atrophy in both groups. Morphometry analyses showed asymmetric right posterolateral atrophy in C9orf72 negative ALS. |
| Convery et al, 2020 (19) | Pre-symptomatic C9orf72 n=73  Symptomatic C9orf72 n=31  Pre-symptomatic GRN n=104  Symptomatic GRN n=24  Pre-symptomatic MAPT n=39  Symptomatic MAPT n=10  Controls n=181 | Cross-sectional  Case-control | N/A | N/A | VBM | Abnormal pain perception in symptomatic C9orf72 was associated with thalamo-cortico-striatal network atrophy involving the posterior thalamus, striatum, orbitofrontal cortices, inferomedial temporal lobes and cerebellum. |
| Cury et al, 2019 (20) | Pre-symptomatic GRN n=53  Pre-symptomatic C9orf72 n=34  Pre-symptomatic MAPT n=26  Controls n= 98 | Cross-sectional  Case control | N/A | N/A | MRI – large diffeomorphic deformation metric mapping | In a group of pre-symptomatic FTD mutation carriers, altered shape of the anterior thalamus was detected at least 5 years before expected symptom onset. |
| De Reuck et al, 2014 (21) | FTLD n=37  AD n=46  ALS n=11  LBD n=13  PSP n=14  VaD n=16  Controls n=15 | Cross-sectional  Case control | All cases | N/A | Quantification of iron deposits noted on MRI GRE sequence | There was significant iron load only in FTLD compared to other neurodegenerative disorders. It particularly involved the claustrum, caudate, putamen, globus pallidus, thalamus and subthalamic nucleus. |
| De Reuck et al, 2017 (22) | ALS n=12  FTLD n=38 (FUS n=6; Tau n=13; TDP n=19)  Controls n=28 | Cross-sectional  Case control | All cases | N/A | Quantification of iron deposits noted on MRI GRE sequence | A significant increase of iron deposition was observed in the thalamus in FTLD-FUS and FTLD-TDP groups. |
| Devenney et al, 2017 (23) | bvFTD n=36; (C9orf72 positive n=9/36)  FTD-ALS n=20 (C9orf72 positive n=5/20)  Controls n=23 | Cross-sectional  Case control | N/A | N/A | VBM | There was bilateral thalamic volume loss in both FTD phenotypes. Amongst other cortical and subcortical areas, thalamic atrophy was associated with increased psychotic symptomatic in C9orf72 mutation carriers. |
| Devenney et al, 2021 (24) | ALS n=28  ALS-Plus n=9  ALS-FTD n=11  bvFTD n=27  Controls n=25 | Cross-sectional  Case-control | N/A | N/A | VBM | Psychosis-like experiences and perceptual abnormalities were associated with frontotemporal, cerebellum and anterior thalamus atrophy. |
| Fletcher et al, 2015 (25) | FTLD n=31  Phenotype: bvFTD n=15; svPPA n=11; nfvPPA n=5  Genotype: sporadic n=24; C9orf72 n=6; MAPT n=2  AD n=20 | Cross-sectional  Case-control | N/A | N/A | VBM | In C9orf72 mutation carriers, posterior thalamic atrophy was associated with pain and temperature symptoms. |
| Garibotto et al, 2011 (26) | bvFTD n=38  svPPA n=9  nfvPPA n=6  Controls n=25 | Cross-sectional  Case-control | N/A | N/A | Subcortical volume | In bvFTD and nfvPPA, there was bilateral thalamic atrophy. No thalamic atrophy was observed in svPPA. |
| Harper et al, 2017 (27) | Dementia n=186 (AD n= 107; DLB n=25; FTLD n=54 [3R-tau n=11; 4R-tau n=17; TDP43A n=12; TDP43C n=14])  Controls n=73 | Cross-sectional  Case-control | All cases | N/A | VBM | The thalamic region was significantly affected in all 3 FTLD pathologies (3R- and 4R-tau, TDP43A and C). |
| Hornberger et al, 2012 (28) | In vivo cohort: bvFTD n=15; AD n=19; controls n=18  Post-mortem cohort: bvFTD n=19; AD n=18; Controls n=20 | Cross-sectional  Case-control | Post-mortem cohort: bvFTD n=19 [TDP type A n=6; TDP type B n=3; Tau n=10 – Picks disease n=7; CBD n=3]; AD n=18; Controls n=20 | N/A | VBM  DTI | In bvFTD, there was atrophy of subcortical Papez circuit regions (fornix and anterior thalamus) in the clinical and post-mortem cohort. There was no difference between TDP-43 and Tau pathology. The extent of anterior thalamus atrophy determines the degree of amnesia. |
| Irwin et al, 2013 (29) | C9orf72 positive n=64 (ALS n=31; FTD n=22 [bvFTD n=17; svPPA n=1; nfvPPA n=4]; ALS-FTD n=9; AD n=2)  C9orf72 negative n=79 (ALS n=36; FTD n=43 [bvFTD n=23; svPPA n=7; CBS n=2]; ALS-FTD n=10; AD n=1) | Cross-sectional  Case-control | C9orf72 positive n=13; and C9orf72 negative n=12 | N/A | VBM | There was greater thalamic atrophy on imaging, and a trend towards greater thalamic neuronal loss and gliosis in C9orf72 positive FTLD versus C9orf72 negative FTLD. |
| Irwin et al, 2016 (30) | Pick’s disease n=21 (bvFTD n=16; nfvPPA+bvFTD n=1; ALS-FTD n=1; CBS n=2; AD n=1)  Controls for imaging n=60 | Cross-sectional  Case series | All cases | N/A | GM density  DTI | Four stages of sequential regional pathological tau deposition were described. The second phase showed subcortical involvement including the thalamus.  Interval MRI scans detected minimal thalamic atrophy. |
| Kumfor et al, 2015 (31) | svPPA n=11  bvFTD n=13  Controls n=11 | Cross-sectional  Case-control | N/A | N/A | VBM | In bvFTD, deficits in object memory were associated with thalamic degeneration – particularly on the left-side. |
| Landin-Romero et al, 2017 (32) | bvFTD n=37  AD n=41  Controls n=33 | Longitudinal  Case-control | N/A | 1-, 2-, 3- and 4-years | Cortical thickness  Subcortical volumes | There was significant progressive volume loss of the thalamus in bvFTD and AD that was greater in bvFTD. |
| Links et al, 2009 (33) | FTD n=21  Controls n=21 | Cross-sectional  Case-series | N/A | N/A | Subcortical volumes | The mean posterior thalamus volume was smaller in those with apathy compared to those without apathy, trending towards statistical significance. The mean anterior thalamus volume was similar in both groups. |
| Machts et al, 2015 (34) | C9orf72 positive ALS n=67 (ALS-FTD n=7; ALS with cognitive/behavioural impairment n=18; and ALS with no cognitive impairment n=42)  Controls n=39 | Cross-sectional  Case-control | N/A | N/A | Subcortical volume, density and shape analyses | In ALS-FTD, there was bilateral thalamic involvement. Thalamic shape analyses indicated pathologic changes in the anterior nuclei, ventral anterior nucleus, the lateral dorsal nuclei and internal medullary nuclei. |
| Mahoney et al, 2011 (35) | svPPA n=43 | Cross-sectional  Case-control | N/A | N/A | VBM | In svPPA with auditory symptoms, there was atrophy of the medial geniculate nucleus in the auditory thalamus. |
| Manera et al, 2019 (36) | bvFTD n=70  Controls n=133 | Longitudinal  Case-control | N/A | 1-year | Deformation-based morphometry analyses | There was statistically significant progressive thalamic atrophy in bvFTD compared with controls. |
| Mann et al, 1993 (37) | FTD n=10  FTD-ALS n=6 | Cross-sectional  Case series | All cases | N/A | Cortical thickness  Cortical and subcortical cross-sectional areas  Cortical ribbon length | Thalamic atrophy was detected in both FTD and FTD-ALS. This was greater in FTD compared to FTD-ALS. |
| McKenna et al, 2021 (38) | bvFTD n=10  nfvPPA n=15  svPPA n=5  C9orf72 positive ALS-FTD n=20  C9orf72 negative ALS-FTD n=20  Controls n=100 | Cross-sectional  Case control | N/A | N/A | Thalamus  Segmentation  Vertex analyses  Morphometry | In [bvFTD](https://www.sciencedirect.com/topics/medicine-and-dentistry/frontal-variant-frontotemporal-dementia), there was widespread bilateral volume loss involving all thalamic nuclei.  In [nfvPPA](https://www.sciencedirect.com/topics/medicine-and-dentistry/primary-progressive-aphasia), there was bilateral anteroventral, mediodorsal, laterodorsal, lateroposterior, lateral and medial geniculate degeneration; and left-sided motor and sensory thalamic nuclei involvement.  In svPPA, there was bilateral mediodorsal, lateral and medial geniculate atrophy; and left-sided anteroventral, laterodorsal, lateroposterior, and pulvinar degeneration.  In [*C9orf72*](https://www.sciencedirect.com/topics/biochemistry-genetics-and-molecular-biology/c9orf72)-positive ALS-FTD, there was bilateral anteroventral, mediodorsal; left laterodorsal, lateroposterior, ‘motor’; and right [pulvinar](https://www.sciencedirect.com/topics/medicine-and-dentistry/pulvinar) degeneration.  In C9orf72-negative ALS-FTD, there was bilateral anteroventral, mediodorsal, motor, lateral and medial [geniculate nuclei](https://www.sciencedirect.com/topics/medicine-and-dentistry/geniculate-nucleus); left lateroposterior, sensory and intralaminar nuclei; and right pulvinar nuclei atrophy |
| McMillan et al, 2015 (39) | C9orf72 mutation carriers n=55  (In vivo n=20; Post-mortem n=35) | Longitudinal (n=11) | Post-mortem C9orf72 mutation carriers n=35 | 1-year | GM density | Preserved GM density in hippocampus, frontal cortex and thalamus were associated with C9orf72 promoter hypermethylation. Longitudinal imaging studies show that C9orf72 promoter hypermethylation is associated with reduced progressive decline in those GM regions. |
| Meysami et al, 2022 (40) | bvFTD n=20  EOAD n=45 | Cross-sectional | N/A | N/A | Volumetric quantification of lobar, subcortical and hippocampal volumes | MR based volumetric quantification percentiles may help differentiate bvFTD vs. EOAD. In bvFTD, there was lower volume in the frontal lobes, thalamus and putamen. |
| Mioshi et al, 2013 (41) | FTD n=52  AD n=20  Controls n=18 | Cross-sectional  Case control | N/A | N/A | VBM | In FTD, there was prefrontal and thalamus atrophy, involving the medial dorsal nucleus that is connected to PFC. It was associated with ADL dysfunction. |
| Möller et al, 2015 (42) | FTD n=24  AD n=72  Controls n=72 | Cross-sectional  Case control | N/A | N/A | Subcortical volume | There was thalamic atrophy in both FTD and AD that differentiated from controls but not between subgroups. |
| Pasquini et al, 2020 (43) | Clinical: bvFTD n=5; bvFTD-ALS n=9; ALS n=2  Pathological: FTLD-TDP-B n=10; FTLD-TDP-U n=3; ALS-TDP n=3  Genetic: C9orf72 positive n=7; C9orf72 negative n=9 | Cross-sectional  Case series | All cases | N/A | MRI - VBM | TDP-43 pathology within the right frontoinsular von Economo neurons and fork cells is associated with salience network atrophy involving the insula, medial frontal and thalamic regions. This was associated with loss of empathy, suggesting that selective network degeneration may explain the behavioural impairment. |
| Popuri et al, 2018 (44) | Pre-symptomatic C9orf72 n=15  Pre-symptomatic GRN n= 9  Controls n=38 | Cross-sectional  Case control | N/A | N/A | Cortical thickness  Subcortical volume | In pre-symptomatic C9orf72, there was cortical thinning in the temporal, parietal and frontal regions. There was reduced volumes of bilateral thalamus and left caudate.  In pre-symptomatic GRN, there was no difference in cortical thickness or subcortical volumes compared with controls. |
| Possin et al, 2012 (45) | MCI n=53  Dementia n=110  (bvFTD n=32; AD n=32; svPPA n=25; nfvPPA n=6; PSP n=10; CBS n=5)  Controls n=37 | Cross-sectional  Case control | N/A | N/A | Volumetry | Design fluency was associated with maintenance of frontal, temporal, parietal, striatal and thalamic volume.  Compared to all dementia subgroups, patients with bvFTD made more repetition errors which were correlated by orbitofrontal cortical atrophy. |
| Rohrer et al, 2010 (46) | FTLD-TDP n=28 (Type 1 n=9; type 2 m=5; type 3 n=10; unspecified n=4)  Controls n=50 | Cross-sectional  Case control | All cases | N/A | VBM | FTLD-TDP-43 subtypes have distinct clinical and neuroimaging features. Thalamic atrophy was detected in type A FTLD-TDP-43. This pathological subtype was associated with bvFTD or FTD-ALS phenotype. |
| Rohrer et al, 2015 (47) | Pre-symptomatic C9orf72 n=18  Symptomatic C9orf72 n=16  Pre-symptomatic MAPT n=15  Symptomatic MAPT n=11  Pre-symptomatic GRN n=45  Symptomatic GRN n=13  Controls n=102 | Cross-sectional  Case control | N/A | N/A | Cortical volume  Subcortical volume | In pre-symptomatic C9orf72, there was involvement of the thalamus, insula and posterior cortical areas at 25 years before expected onset; followed by frontal and temporal lobes at 20 years before expected onset, and cerebellum 10-years before symptom onset. |
| San Lee et al, 2020 (48) | nfvPPA n=38 (age <65-years n=14; age >65-years n=24)  Controls n=76 | Cross-sectional  Case control | N/A | N/A | Cortical thickness  Subcortical shape and volume analysis | In nfvPPA, there was abnormal surface shape of subcortical structures involving left striatum, thalamus, hippocampus and amygdala in those aged <65-years; and left striatum and thalamus in those aged >65-years. |
| Seeley et al, 2008 (49) | bvFTD n=45  Controls n=45 | Cross-sectional  Case control | N/A | N/A | VBM | In those with very mild bvFTD, there was GM atrophy involving the dorsomedial thalamus, that became more diffuse and widespread with progression of the disease. |
| Sellami et al, 2018 (50) | Familial FTD mutation carriers n=167 (GRN n=75; C9orf72 n=60; MAPT n=32) | Cross-sectional  Case control | N/A | N/A | VBM | In GRN mutation carriers, psychotic symptoms (delusions and hallucinations) correlated with anterior insula, left thalamus, cerebellum and cortical atrophy. |
| Sha et al, 2012 (51) | C9orf72 mutation carriers n=31 (bvFTD n=15; FTD-ALS n=11; ALS n=5)  Sporadic disease controls n=73 (bvFTD n=48, FTD-ALS n=19; ALS n=6) | Cross-sectional  Disease control | N/A | N/A | VBM | There was greater thalamic atrophy in C9orf72 mutation carriers with bvFTD (bilateral; L>R) and FTD-ALS (right thalamus) phenotypes compared to non-carriers. |
| Spinelli et al, 2021(52) | Genetic FTLD n=66  (bvFTD n=12; bvFTD-ALS n=5; nfvPPA n=3; svPPA n=2; ALS n=35; PMA n=6; PLS n=3)  (C9orf72 n=33; TARDBPn=10; SOD1 n=7; GRN n=8; FUS n=2; TBK2 n=2; MAPT n=1; TREM2 n=1; C9orf72+GRN n=1; C9orf72+TARDBP n=1)  Sporadic FTLD n=61  (bvFTD n=12; nfvPPA n=2; svPPA n=2; ALS n=37; PMA n=5; PLS n=3) | Cross-sectional  Case control | N/A | N/A | VBM | There was greater bilateral thalamic atrophy in genetic FTLD compared with sporadic FTLD, with widespread involvement in C9orf72 mutation carriers and selective anterior thalamic involvement in GRN mutation carriers. |
| Sturm et al, 2017 (53) | bvFTD n=20  AD n=15  Controls n=39 | Cross-sectional  Case control | N/A | N/A | VBM | Prosocial giving was lower in bvFTD and associated with lateralised atrophy in the reward-relevant system involving the right pulvinar nucleus of the thalamus. |
| Sturm et al, (54) 2018 | bvFTD n=30  AD n=25  Controls n=25 | Cross-sectional  Case control | N/A | N/A | VBM | In bvFTD, left-lateralised salience network and bilateral medial pulvinar thalamic nuclei atrophy were associated with impaired behaviour e.g. less empathy, patience, engagement and motivation to help those in need |
| Toller et al, 2020 (55) | Controls n=140  Pre-symptomatic genetic FTD n=71  Behavioural genetic MCI n=12  (C9orf72 n=5; MAPT n=3; GRN n=4)  Sporadic bvFTD n=154  Genetic bvFTD n=71; (C9orf72 n=36, MAPT n=26, GRN n=9) | Longitudinal (n=62: behavioural genetic MCI n=7; sporadic bvFTD n=35; genetic bvFTD n=20; controls n=53)  Case control | N/A | Not specified | VBM | The revised self-monitoring scale, an informant measure of socioemotional sensitivity, predicted thalamic volume in very mild and mild disease stages of bvFTD. |
| van der Burgh et al, 2020 (56) | ALS n=292 (C9orf72 mutation carriers n=24)  Controls n=156 | Longitudinal (ALS n=150; controls n=72) | N/A | ALS C9orf72 negative n=133; 5.3 (4.2-6.4) months)  ALS C9orf72 positive n=17; 5.1 (3.7 – 6.4) months | Cortical thickness  Subcortical volumes  DTI | In C9orf72 positive ALS, there was reduced bilateral thalami volume. In C9orf72 negative ALS and in those with ALS and impaired behaviour there was reduced right thalamus volume. There was no change in thalamus volume in those with ALS and impaired cognition. |

**Supplementary Table 3**. White matter imaging

| **Diffusion imaging and white matter analyses** | | | | | | |
| --- | --- | --- | --- | --- | --- | --- |
| **First author, year of publication** | **Patient groups and cohort sizes** | **Study design** | **Neuropathology** | **Follow-up duration** | **Imaging modality; single; multiple; whole or ROI** | **Key outcomes** |
| Bede et al, 2013 (57) | C9orf72 positive ALS n=9 (ALS-FTD n=6; ALS with executive dysfunction n=2)  C9orf72 positive ALS n=30 (ALS-FTD n=3; ALS with executive dysfunction n=2)  Controls n=40 | Cross-sectional  Case-control | N/A | N/A | VBM  Cortical thickness  DTI | In a combined group of C9orf72 positive ALS with and without cognitive impairment or comorbid FTD, there was loss of white matter integrity affecting the bilateral thalami. |
| Bertrand et al, 2019 (58) | Pre-symptomatic C9orf72 n= 41  Control n=39 | Cross-sectional  Case control | N/A | N/A | Cortical volumes  Subcortical volumes  DTI | In pre-symptomatic C9orf72, there was right thalamus atrophy and right anterior thalamic radiations involvement. |
| Daianu et al, 2016 (59) | bvFTD n=20  EOAD n=23  Controls n=33 | Cross-sectional  Case control | N/A | N/A | DTI | In bvFTD, there was widespread loss of WM integrity – especially involving the bilateral uncinate fasciculus, frontal callosum, anterior thalamic radiations, cingulum bundles and left superior longitudinal fasciculus. |
| Downey et al, 2015 (60) | bvFTD n=29  svPPA n=15  Controls n=37 | Cross-sectional  Case control | N/A | N/A | VBM  DTI | In svPPA and bvFTD, impaired social cognition was associated with involvement of the WM tracts: fornix, uncinate fasciculus and anterior thalamic radiation. |
| Feis et al, 2019 (61) | Pre-symptomatic GRN n=28  Pre-symptomatic MAPT n=11  Controls = 36 | Cross-sectional  Case control | N/A | N/A | VBM  DTI  rs-fMRI | No difference in GM volume, DTI metrics or functional connectivity in combined pre-symptomatic GRN/MAPT mutation carriers compared to controls including ROI analyses of the anterior thalamic radiation. |
| Floeter et al, 2016 (62) | C9orf72 mutation carriers n =28 (Asymptomatic n=7; ALS n=11; ALS-FTD n=7; bvFTD n=3)  Controls n=28 | Longitudinal (n=20)  Case control | N/A | Initial follow-up scan in 6-months (n=19) and 18-months (n=1); and interval scan of the former group in 18-months (n=11) | TBSS | There was widespread loss of WM integrity in C9orf72 mutation carriers, including the anterior thalamic radiation |
| Jakabek et 2018 (63) | bvFTD n=24  Controls n=24 | Cross-sectional  Case control | N/A | N/A | Cortical volumes  Subcortical volumes  DTI | In bvFTD, there were disruptions in the frontal-striatal-thalamic pathways. The thalamus and striatum were smaller in regions linked to the dorsolateral prefrontal cortex; unexpectedly larger in regions linked to the medial prefrontal cortex, perhaps compensatory; and inconsistent findings in regions linked to the orbitofrontal cortex. |
| Mahoney et al, 2012 (64) | C9orf72 mutation carriers n=19 (bvFTD n=12; FTD-ALS n=3; nfvPPA n=1; not specified n=3) | Cross-sectional  Case series | N=6 | N/A | VBM  Volumetry  Cortical thickness  DTI | There was bilateral thalamic atrophy and disrupted anterior thalamic radiations in C9orf72 mutation carriers. |
| Masuda et al, 2016 (65) | ALS with impaired cognition n=25  ALS-FTD n=7  ALS with normal cognition n=19 | Cross-sectional  Case control | N/A | N/A | VBM  DTI | Thalamic atrophy was detected in ALS-FTD, but not cognitively impaired ALS or normal cognition ALS.  Loss of integrity of the superior thalamic radiation was described in cognitively impaired ALS and ALS-FTD. |
| Möller et al, 2015 (66) | AD n=39  bvFTD n=30  Controls n=41 | Cross-sectional  Case control | N/A | N/A | VBM  Subcortical segmentation  DTI | There was thalamic atrophy in both AD and bvFTD, that discriminated those with dementia from controls.  In bvFTD, there was also loss of WM integrity involving the thalamus and anterior thalamic radiation. |
| Panman et al, 2019 (67) | Pre-symptomatic GRN n=33  Pre-symptomatic MAPT n=15  Pre-symptomatic C9orf72 n=12  Controls n=53 | Longitudinal  Case-control | N/A | 2-years | VBM  Cortical thickness  TBSS | In pre-symptomatic C9orf72 mutation carriers, there was thalamic atrophy compared to controls, MAPT and GRN mutation carriers; and loss of WM integrity involving the bilateral anterior thalamic radiation compared to controls and MAPT but not GRN mutation carriers.  In MAPT mutation carriers, there was loss of integrity of the left anterior thalamic radiation compared to controls. |
| Pampa et al, 2017 (68) | Pre-symptomatic C9orf72 n=18  Control n=15 | Cross-sectional  Case control | N/A | N/A | VBM  DTI | In pre-symptomatic *C9orf72*, there was loss of integrity of anterior thalamic and other WM tracts that were associated with executive dysfunction. In a subgroup of those aged >40 years, there was left thalamic atrophy. |
| Spotorno et al, 2020 (69) | bvFTD n=20  Controls n=22 | Cross-sectional  Case control | N/A | N/A | VBM  TBSS | The increased serum neurofilament light-chain was associated with reduced fractional anisotropy in several WM tracts including the anterior thalamic radiation. |

**Supplementary Table 4**. Functional MRI

| **fMRI** | | | | | | |
| --- | --- | --- | --- | --- | --- | --- |
| **First author, year of publication** | **Patient groups and cohort sizes** | **Study design** | **Neuropathology** | **Follow-up duration** | **Imaging modality; single; multiple; whole or ROI** | **Key outcomes** |
| Agosta et al, 2017 (70) | C9orf72 positive n=19  Sporadic ALS n=29  Sporadic cognitively impaired ALS n=24  Sporadic early onset ALS n=14  Healthy controls n=22 | Cross-sectional  Case-control | N/A | N/A | Cortical thickness  Subcortical volume  DTI  fMRI | In C9orf72 mutation carriers, there was right-sided thalamic atrophy compared to controls and all sporadic cases. |
| Dopper et al, 2016 (71) | Pre-symptomatic MAPT n = 11  Pre-symptomatic GRN n = 23  Controls n=31 | Longitudinal  Case-control | N/A | Mean 2.2 (2.1-2.3) years | Arterial spin labelling | There was frontoparietal and thalamic hypoperfusion in a subgroup of pre-symptomatic GRN mutation carriers. |
| Farb et al, 2013 (72) | bvFTD n=8  svPPA n=8  Controls n=16 | Cross-sectional  Case-control | N/A | N/A | fMRI – independent component analysis | In bvFTD and svPPA, limbic connectivity was reduced in the insula, putamen, anterior thalamus and middle cingulate cortex; and elevated in the PFC. The reduced connectivity in the anterior thalamus and elevated connectivity in the PFC were associated with greater levels of apathy. |
| Lee et al, 2014 (73) | C9orf72 positive n=14 (bvFTD n=9; FTD-ALS n=5)  C9orf72 negative n=14 (bvFTD n=9; FTD-ALS n=5)  Controls n=14 | Cross-sectional  Case-control | N/A | N/A | MRI – VBM  fMRI – Intrinsic connectivity network integrity analyses | There was bilateral thalamic atrophy in sporadic and genetic FTD. It was more marked in C9orf72 mutation carriers, where it particularly involved the left medial pulvinar thalamic nucleus and correlated with reduced salience network connectivity. |
| Lee et al, 2017 (74) | Pre-symptomatic C9orf72 n=15  Control n=15 | Cross-sectional  Case control | N/A | N/A | VBM  DTI  fMRI - Intrinsic connectivity network integrity analyses | In pre-symptomatic C9orf72, there was thalamic atrophy, mainly in the medial pulvinar thalamus; and intrinsic connectivity network deficits that were more prominent in salience and medial pulvinar thalamus-seeded networks. |
| Lee et al, 2019 (75) | Pre-symptomatic GRN n=14  Pre-clinical GRN n=3  Controls n=30 | Cross-sectional  Case control | N/A | N/A | VBM  fMRI - Intrinsic connectivity network integrity analyses | In a group of pre-symptomatic and pre-clinical GRN, there was markedly enhanced connectivity in the salience, nfvPPA, CBS and default mode network. Thalamo-cortical network hyperconnectivity was the unifying feature. |
| Ng et al, 2021 (76) | bvFTD n=14  AD n=50  Controls n=47 | Cross-sectional  Case control | N/A | N/A | rs-fMRI | bvFTD exhibited disrupted integration of the salience network, with low nodal efficiency within the thalamus. |
| Rijpma et al, 2022 (77) | bvFTD n=44  Controls n=44 | Cross-sectional  Case control | N/A | N/A | fMRI - Intrinsic connectivity network integrity analyses | Disrupted salience network connectivity, especially periaqueductal gray output to cortical and thalamic nodes, was associated with social behaviour deficits in bvFTD. |
| Rombouts et al, 2003 (78) | FTD n=7  AD n=7 | Cross-sectional  Case control | N/A | N/A | rs-fMRI | The working memory network in FTD and AD included frontal and parietal lobe and thalamus. In early FTD, the functional changes preceded the structural changes. |
| Shoukry et al, 2020 (79) | Pre-symptomatic C9orf72 n=15  Symptomatic C9orf72 n=27  Controls n=48 | Longitudinal  Case-control | N/A | 6-months  18-months | MRI – rs-fMRI | In pre-symptomatic and symptomatic C9orf72 mutation carriers, there was reduced connectivity in the thalamic network that remained stable compared to controls. |
| Toller et al, 2018 (80) | Neurodegenerative disorder n=103  (bvFTD n=14; AD n=29; PSP n=20; svPPA n=21 and nfvPPA n=19)  Controls n=65 | Cross-sectional  Case control | N/A | N/A | fMRI - Intrinsic connectivity network integrity analyses | The higher functional connectivity in the salience network, particularly between the right anterior insula and cortical and subcortical nodes including the dorsomedial thalamus, predicted socioemotional sensitivity in controls showing that it is a behavioural marker of salience network function.  In bvFTD, there was a trend towards reduced salience network connectivity, but it did not reach statistical significance, perhaps because of small sample size. |
| Zhou et al, 2010 (81) | bvFTD n=12  AD n=12  Controls n=12 | Cross-sectional  Case control | N/A | N/A | fMRI - Intrinsic connectivity network integrity analyses | In bvFTD, there was salience network disruption involving the frontoinsular, cingulate, striatal, thalamic and brainstem nodes. Default mode network was enhanced. |

**Supplementary Table 5**. Positron emission tomography

| **PET** | | | | | | |
| --- | --- | --- | --- | --- | --- | --- |
| **First author, year of publication** | **Patient groups and cohort sizes** | **Study design** | **Neuropathology** | **Follow-up duration** | **Imaging modality** | **Key outcomes** |
| Cistaro et al, 2014 (82) | C9orf72 positive ALS n=15  Sporadic ALS-FTD n=12  Sporadic ALS without cognitive impairment n=30 | Cross-sectional  Case-Control | N/A | N/A | [18F] FDG-PET | In comparison with sporadic ALS, C9orf72 positive ALS was associated with hypometabolism in the left frontal, superior temporal, anterior and posterior cingulate cortex, insula, caudate and thalamus; and hypermetabolism in the midbrain, bilateral occipital cortex, globus pallidus and left inferior temporal cortex. |
| De Vocht et al, 2020 (83) | Pre-symptomatic C9orf72 n = 17  Controls n=25 | Cross-sectional  Case-Control | N/A | N/A | [18F] FDG-PET | In pre-symptomatic C9orf72, there was relative hypometabolism in the frontotemporal regions, basal ganglia and thalami; and relative hypermetabolism in the pre-central gyrus and precuneus cortex. These abnormalities may be detected before elevated CSF neurofilament light chains. |
| Diehl-Schmid et al, 2007 (84) | bvFTD n=22  Controls n=15 | Longitudinal  Case-Control | N/A | 19.5 months | [18F] FDG-PET | In bvFTD, there was significant symmetrical hypometabolism of the frontal lobes, insula, caudate and thalamus bilaterally. This was followed by interval progression of hypometabolism to involve the parietal and temporal cortices. |
| Diehl-Schmid et al, 2019 (85) | FTLD C9orf72 mutation carriers n=22  FTLD non-mutation carriers n=22  Controls n=23 | Cross-sectional  Case-Control | N/A | N/A | [18F] FDG-PET | Compared to non-carriers, there was marked bilateral thalamic hypometabolism in FTLD C9orf72 mutation carriers. Right-sided thalamic hypometabolism was detected in FTLD non-carriers compared to controls. It did not correlate with disease severity, duration or psychotic symptoms. |
| Frisch et al, 2013 (86) | FTLD n=11 (svPPA n=5; bvFTD N=4; mixed n=2)  AD n=19  Controls n=13 | Cross-sectional  Case-Control | N/A | N/A | VBM  [18F] FDG-PET | In both groups, there was hippocampus and thalamus atrophy; and only in the FTD subgroup, there was insula, basal ganglia and basal forebrain atrophy.  In FTD, impaired immediate recall of visual information was correlated with thalamic atrophy, amongst other regions: bilateral inferior frontal, orbitofrontal, parahippocampal, basal ganglia, and subcallosal regions. |
| Grimmer et al, 2004 (87) | FTD n=10  Controls = not specified | Longitudinal  Case-Control | N/A | 17.1$\pm$6.0 months | [18F] FDG-PET | In FTD, there was relative hypometabolism in frontal cortical areas, caudate and thalami with interval progression in orbitofrontal and subcortical regions. |
| Ishii et al, 1998 (88) | FTD n=21  AD n=21  Controls n=21 | Cross-sectional  Case-Control | N/A | N/A | [18F] FDG-PET | In comparison with AD, there was marked hypometabolism in thalami in FTD. |
| Jang et al, 2018 (89) | FTD n=4 (bvFTD n=2; nfvPPA n=1; svPPA n=1)  AD n=2  Controls n=2 | Cross-sectional  Case-Control | N/A | N/A | MRI  [^18^F]-Florbetaben amyloid PET  THK5351 and AV-1451 tau PET | Regardless of the subtype of dementia, there was ‘off-target’ binding of tau tracers AV-1451 and THK5351 in the WM, midbrain, thalamus and basal ganglia. The latter had greater ‘off-target’ binding that was more prominent in FTD. This suggests that AV-1451 is more sensitive and specific to AD-type tau, and that THK5351 may indicate more non-specific neurodegeneration. |
| Jeong et al, 2005 (90) | FTD n=29  Controls n=11 | Cross-sectional  Case-Control | N/A | N/A | [18F] FDG-PET | Amongst other cortical and subcortical regions, there was hypometabolism involving the medial thalamic structures in combined group of FTD phenotypes. |
| Leuzy et al, 2015 (91) | bvFTD n=5  Controls n=10 | Cross-sectional  Case-Control | N/A | N/A | VBM  [18F] FDG-PET  [(11)C]ABP688) PET | In bvFTD, there was decreased [(11)C]ABP688) L>R thalamus binding, left thalamus hypometabolism and bilateral thalamic volume loss. There was overlap of thalamic involvement across these three imaging modalities. |
| Malpetti et al, 2021(92) | Pre-symptomatic C9orf72 n = 3  Symptomatic C9orf72 n=1  Controls n= 19 | Cross-sectional  Case-Control | N/A | N/A | MRI  [^11^ C]UCB-J PET | There was pre-symptomatic loss of synaptic density that is most marked in the pulvinar and ventral-posterior thalamic subregions compared to controls with progression involving the frontotemporal regions in symptomatic patient. |
| Matias-Guiu et al, 2015 (93) | FTD n=33  AD n=33  Other diagnoses n=33 | Cross-sectional  Case-Control | N/A | N/A | [18F] FDG-PET | The re-emergence of primitive reflexes was more commonly observed in FTD compared to AD and other diagnoses. It was associated with hypometabolism involving the bilateral superior frontal gyri, bilateral putamina and thalami. |
| Poljansky et al, 2011 (94) | FTLD n=16 (bvFTD n=9; nfvPPA n=4; svPPA n=3)  AD n=16  MCI n=11 | Cross-sectional  Case-Control | N/A | N/A | [18F] FDG-PET | There was mild thalamic hypometabolism in 3 patients with FTLD and moderate thalamic hypometabolism in 2 patients with FTLD. |
| Popuri et al, 2021 (95) | Pre-symptomatic C9orf72 n=15  Controls n=20 | Cross-sectional | N/A | N/A | MRI - Volumetry  FDG-PET | In pre-symptomatic C9orf72, there was hypometabolism of thalami, cingulate gyrus, frontal and temporal cortices, up to 10-years before symptom onset, that preceded GM volume loss. |
| Schaeverbeke et al, 2018 (96) | PPA n=20 (nfvPPA n=12; svPPA n=5; lvPPA n=3)  Controls n=64 | Cross-sectional  Case-Control | N/A | N/A | MRI – VBM  [^18^F]-THK5351 PET   [^11^C]-Pittsburgh Compound B PET | Elevated tau-tracer [^18^F]-THK5351 binding was detected in the thalamus in mixed, non-fluent and semantic variant PPA. In particular, the pattern of thalamic involvement observed in mixed PPA differed from lvPPA. |
| Soleimani-Meigooni et al, 2020 (97) | AD n=8  FTLD Tauopathies n=9 (PSP n=4; CBD n=2; MAPT n=2; AGD n=1)  FTLD Non-Tauopathies n=3 (GRN n=1; C9orf72 n=1; FUS n=1) | Cross-sectional | All cases | N/A | MRI  18F-flortaucipir PET | In the single case of C9orf72 positive FTLD, there was increased tracer uptake in the frontal white matter, striatum, thalamus, substantia nigra, and pons. At autopsy, there was some tau pathology and extensive TDP-43 inclusions in the frontal lobes, and only TDP-43 in the striatum, thalamus, and substantia nigra. |

**References**

1. Brettschneider J, Del Tredici K, Irwin DJ, Grossman M, Robinson JL, Toledo JB, et al. Sequential distribution of pTDP-43 pathology in behavioral variant frontotemporal dementia (bvFTD). Acta Neuropathologica. 2014;127(3):423-39.

2. Kawles A, Nishihira Y, Feldman A, Gill N, Minogue G, Keszycki R, et al. Cortical and subcortical pathological burden and neuronal loss in an autopsy series of FTLD-TDP-type C. Brain. 2022;145(3):1069-78.

3. Yang Y, Halliday GM, Hodges JR, Tan RH. von Economo Neuron Density and Thalamus Volumes in Behavioral Deficits in Frontotemporal Dementia Cases with and without a C9ORF72 Repeat Expansion. J Alzheimers Dis. 2017;58(3):701-9.

4. Ahmed RM, Irish M, Henning E, Dermody N, Bartley L, Kiernan MC, et al. Assessment of Eating Behavior Disturbance and Associated Neural Networks in Frontotemporal Dementia. JAMA Neurol. 2016;73(3):282-90.

5. Ahmed RM, Landin-Romero R, Liang CT, Keogh JM, Henning E, Strikwerda-Brown C, et al. Neural networks associated with body composition in frontotemporal dementia. Ann Clin Transl Neurol. 2019;6(9):1707-17.

6. Ahmed RM, Bocchetta M, Todd EG, Tse NY, Devenney EM, Tu S, et al. Tackling clinical heterogeneity across the amyotrophic lateral sclerosis-frontotemporal dementia spectrum using a transdiagnostic approach. Brain Commun. 2021;3(4):fcab257.

7. Bede P, Omer T, Finegan E, Chipika RH, Iyer PM, Doherty MA, et al. Connectivity-based characterisation of subcortical grey matter pathology in frontotemporal dementia and ALS: a multimodal neuroimaging study. Brain Imaging Behav. 2018;12(6):1696-707.

8. Bocchetta M, Gordon E, Cardoso MJ, Modat M, Ourselin S, Warren JD, et al. Thalamic atrophy in frontotemporal dementia - Not just a C9orf72 problem. Neuroimage Clin. 2018;18:675-81.

9. Bocchetta M, Iglesias JE, Russell LL, Greaves CV, Marshall CR, Scelsi MA, et al. Segmentation of medial temporal subregions reveals early right-sided involvement in semantic variant PPA. Alzheimers Res Ther. 2019;11(1):41.

10. Bocchetta M, Iglesias Espinosa MDM, Lashley T, Warren JD, Rohrer JD. In vivo staging of frontotemporal lobar degeneration TDP-43 type C pathology. Alzheimer's research & therapy. 2020;12(1):34-.

11. Bocchetta M, Iglesias JE, Neason M, Cash DM, Warren JD, Rohrer JD. Thalamic nuclei in frontotemporal dementia: Mediodorsal nucleus involvement is universal but pulvinar atrophy is unique to C9orf72. Hum Brain Mapp. 2020;41(4):1006-16.

12. Bocchetta M, Todd EG, Peakman G, Cash DM, Convery RS, Russell LL, et al. Differential early subcortical involvement in genetic FTD within the GENFI cohort. NeuroImage: Clinical. 2021;30:102646.

13. Branco LMT, de Rezende TJR, Roversi CdO, Zanao T, Casseb RF, de Campos BM, et al. Brain signature of mild stages of cognitive and behavioral impairment in amyotrophic lateral sclerosis. Psychiatry Research: Neuroimaging. 2018;272:58-64.

14. Cajanus A, Katisko K, Kontkanen A, Jääskeläinen O, Hartikainen P, Haapasalo A, et al. Serum neurofilament light chain in FTLD: association with C9orf72, clinical phenotype, and prognosis. Annals of clinical and translational neurology. 2020;7(6):903-10.

15. Cardenas VA, Boxer AL, Chao LL, Gorno-Tempini ML, Miller BL, Weiner MW, et al. Deformation-based morphometry reveals brain atrophy in frontotemporal dementia. Archives of neurology. 2007;64(6):873-7.

16. Cash DM, Bocchetta M, Thomas DL, Dick KM, van Swieten JC, Borroni B, et al. Patterns of gray matter atrophy in genetic frontotemporal dementia: results from the GENFI study. Neurobiology of aging. 2018;62:191-6.

17. Chang JL, Lomen-Hoerth C, Murphy J, Henry RG, Kramer JH, Miller BL, et al. A voxel-based morphometry study of patterns of brain atrophy in ALS and ALS/FTLD. Neurology. 2005;65(1):75-80.

18. Chipika RH, Finegan E, Li Hi Shing S, McKenna MC, Christidi F, Chang KM, et al. "Switchboard" malfunction in motor neuron diseases: Selective pathology of thalamic nuclei in amyotrophic lateral sclerosis and primary lateral sclerosis. Neuroimage Clin. 2020;27:102300.

19. Convery RS, Bocchetta M, Greaves CV, Moore KM, Cash DM, Van Swieten J, et al. Abnormal pain perception is associated with thalamo-cortico-striatal atrophy in &lt;em&gt;C9orf72&lt;/em&gt; expansion carriers in the GENFI cohort. Journal of Neurology, Neurosurgery &amp;amp; Psychiatry. 2020;91(12):1325.

20. Cury C, Durrleman S, Cash DM, Lorenzi M, Nicholas JM, Bocchetta M, et al. Spatiotemporal analysis for detection of pre-symptomatic shape changes in neurodegenerative diseases: Initial application to the GENFI cohort. NeuroImage. 2019;188:282-90.

21. De Reuck JL, Deramecourt V, Auger F, Durieux N, Cordonnier C, Devos D, et al. Iron deposits in post-mortem brains of patients with neurodegenerative and cerebrovascular diseases: a semi-quantitative 7.0 T magnetic resonance imaging study. Eur J Neurol. 2014;21(7):1026-31.

22. De Reuck J, Devos D, Moreau C, Auger F, Durieux N, Deramecourt V, et al. Topographic distribution of brain iron deposition and small cerebrovascular lesions in amyotrophic lateral sclerosis and in frontotemporal lobar degeneration: a post-mortem 7.0-tesla magnetic resonance imaging study with neuropathological correlates. Acta Neurol Belg. 2017;117(4):873-8.

23. Devenney EM, Landin-Romero R, Irish M, Hornberger M, Mioshi E, Halliday GM, et al. The neural correlates and clinical characteristics of psychosis in the frontotemporal dementia continuum and the C9orf72 expansion. NeuroImage: Clinical. 2017;13:439-45.

24. Devenney EM, Tu S, Caga J, Ahmed RM, Ramsey E, Zoing M, et al. Neural mechanisms of psychosis vulnerability and perceptual abnormalities in the ALS-FTD spectrum. Ann Clin Transl Neurol. 2021;8(8):1576-91.

25. Fletcher PD, Downey LE, Golden HL, Clark CN, Slattery CF, Paterson RW, et al. Pain and temperature processing in dementia: a clinical and neuroanatomical analysis. Brain. 2015;138(Pt 11):3360-72.

26. Garibotto V, Borroni B, Agosti C, Premi E, Alberici A, Eickhoff SB, et al. Subcortical and deep cortical atrophy in Frontotemporal Lobar Degeneration. Neurobiol Aging. 2011;32(5):875-84.

27. Harper L, Bouwman F, Burton EJ, Barkhof F, Scheltens P, O'Brien JT, et al. Patterns of atrophy in pathologically confirmed dementias: a voxelwise analysis. Journal of neurology, neurosurgery, and psychiatry. 2017;88(11):908-16.

28. Hornberger M, Wong S, Tan R, Irish M, Piguet O, Kril J, et al. In vivo and post-mortem memory circuit integrity in frontotemporal dementia and Alzheimer’s disease. Brain. 2012;135(10):3015-25.

29. Irwin DJ, McMillan CT, Brettschneider J, Libon DJ, Powers J, Rascovsky K, et al. Cognitive decline and reduced survival in C9orf72 expansion frontotemporal degeneration and amyotrophic lateral sclerosis. J Neurol Neurosurg Psychiatry. 2013;84(2):163-9.

30. Irwin DJ, Brettschneider J, McMillan CT, Cooper F, Olm C, Arnold SE, et al. Deep clinical and neuropathological phenotyping of Pick disease. Ann Neurol. 2016;79(2):272-87.

31. Kumfor F, Hutchings R, Irish M, Hodges JR, Rhodes G, Palermo R, et al. Do I know you? Examining face and object memory in frontotemporal dementia. Neuropsychologia. 2015;71:101-11.

32. Landin-Romero R, Kumfor F, Leyton CE, Irish M, Hodges JR, Piguet O. Disease-specific patterns of cortical and subcortical degeneration in a longitudinal study of Alzheimer's disease and behavioural-variant frontotemporal dementia. Neuroimage. 2017;151:72-80.

33. Links KA, Chow TW, Binns M, Freedman M, Stuss DT, Scott CJ, et al. Apathy is not associated with basal ganglia atrophy in frontotemporal dementia. Am J Geriatr Psychiatry. 2009;17(9):819-21.

34. Machts J, Loewe K, Kaufmann J, Jakubiczka S, Abdulla S, Petri S, et al. Basal ganglia pathology in ALS is associated with neuropsychological deficits. Neurology. 2015;85(15):1301-9.

35. Mahoney CJ, Rohrer JD, Goll JC, Fox NC, Rossor MN, Warren JD. Structural neuroanatomy of tinnitus and hyperacusis in semantic dementia. J Neurol Neurosurg Psychiatry. 2011;82(11):1274-8.

36. Manera AL, Dadar M, Collins DL, Ducharme S. Deformation based morphometry study of longitudinal MRI changes in behavioral variant frontotemporal dementia. Neuroimage Clin. 2019;24:102079.

37. Mann DM, South PW. The topographic distribution of brain atrophy in frontal lobe dementia. Acta Neuropathol. 1993;85(3):334-40.

38. McKenna MC, Li Hi Shing S, Murad A, Lope J, Hardiman O, Hutchinson S, et al. Focal thalamus pathology in frontotemporal dementia: Phenotype-associated thalamic profiles. J Neurol Sci. 2022;436:120221.

39. McMillan CT, Russ J, Wood EM, Irwin DJ, Grossman M, McCluskey L, et al. &lt;em&gt;C9orf72&lt;/em&gt; promoter hypermethylation is neuroprotective. Neurology. 2015;84(16):1622.

40. Meysami S, Raji CA, Mendez MF. Quantified Brain Magnetic Resonance Imaging Volumes Differentiate Behavioral Variant Frontotemporal Dementia from Early-Onset Alzheimer's Disease. J Alzheimers Dis. 2022;87(1):453-61.

41. Mioshi E, Hodges JR, Hornberger M. Neural correlates of activities of daily living in frontotemporal dementia. J Geriatr Psychiatry Neurol. 2013;26(1):51-7.

42. Möller C, Dieleman N, van der Flier WM, Versteeg A, Pijnenburg Y, Scheltens P, et al. More atrophy of deep gray matter structures in frontotemporal dementia compared to Alzheimer's disease. J Alzheimers Dis. 2015;44(2):635-47.

43. Pasquini L, Nana AL, Toller G, Brown JA, Deng J, Staffaroni A, et al. Salience Network Atrophy Links Neuron Type-Specific Pathobiology to Loss of Empathy in Frontotemporal Dementia. Cereb Cortex. 2020;30(10):5387-99.

44. Popuri K, Dowds E, Beg MF, Balachandar R, Bhalla M, Jacova C, et al. Gray matter changes in asymptomatic C9orf72 and GRN mutation carriers. NeuroImage: Clinical. 2018;18:591-8.

45. Possin KL, Chester SK, Laluz V, Bostrom A, Rosen HJ, Miller BL, et al. The frontal-anatomic specificity of design fluency repetitions and their diagnostic relevance for behavioral variant frontotemporal dementia. J Int Neuropsychol Soc. 2012;18(5):834-44.

46. Rohrer JD, Geser F, Zhou J, Gennatas ED, Sidhu M, Trojanowski JQ, et al. TDP-43 subtypes are associated with distinct atrophy patterns in frontotemporal dementia. Neurology. 2010;75(24):2204-11.

47. Rohrer JD, Nicholas JM, Cash DM, van Swieten J, Dopper E, Jiskoot L, et al. Presymptomatic cognitive and neuroanatomical changes in genetic frontotemporal dementia in the Genetic Frontotemporal dementia Initiative (GENFI) study: a cross-sectional analysis. The Lancet Neurology. 2015;14(3):253-62.

48. San Lee J, Yoo S, Park S, Kim HJ, Park K-C, Seong J-K, et al. Differences in neuroimaging features of early- versus late-onset nonfluent/agrammatic primary progressive aphasia. Neurobiology of Aging. 2020;86:92-101.

49. Seeley WW, Menon V, Schatzberg AF, Keller J, Glover GH, Kenna H, et al. Dissociable intrinsic connectivity networks for salience processing and executive control. J Neurosci. 2007;27(9):2349-56.

50. Sellami L, Bocchetta M, Masellis M, Cash DM, Dick KM, van Swieten J, et al. Distinct Neuroanatomical Correlates of Neuropsychiatric Symptoms in the Three Main Forms of Genetic Frontotemporal Dementia in the GENFI Cohort. J Alzheimers Dis. 2018;65(1):147-63.

51. Sha SJ, Takada LT, Rankin KP, Yokoyama JS, Rutherford NJ, Fong JC, et al. Frontotemporal dementia due to C9ORF72 mutations: clinical and imaging features. Neurology. 2012;79(10):1002-11.

52. Spinelli EG, Ghirelli A, Basaia S, Cividini C, Riva N, Canu E, et al. Structural MRI Signatures in Genetic Presentations of the Frontotemporal Dementia/Motor Neuron Disease Spectrum. Neurology. 2021;97(16):e1594-e607.

53. Sturm VE, Perry DC, Wood K, Hua AY, Alcantar O, Datta S, et al. Prosocial deficits in behavioral variant frontotemporal dementia relate to reward network atrophy. Brain Behav. 2017;7(10):e00807.

54. Sturm VE, Sible IJ, Datta S, Hua AY, Perry DC, Kramer JH, et al. Resting parasympathetic dysfunction predicts prosocial helping deficits in behavioral variant frontotemporal dementia. Cortex. 2018;109:141-55.

55. Toller G, Ranasinghe K, Cobigo Y, Staffaroni A, Appleby B, Brushaber D, et al. Revised Self-Monitoring Scale: A potential endpoint for frontotemporal dementia clinical trials. Neurology. 2020;94(22):e2384-e95.

56. van der Burgh HK, Westeneng HJ, Walhout R, van Veenhuijzen K, Tan HHG, Meier JM, et al. Multimodal longitudinal study of structural brain involvement in amyotrophic lateral sclerosis. Neurology. 2020;94(24):e2592-e604.

57. Bede P, Bokde AL, Byrne S, Elamin M, McLaughlin RL, Kenna K, et al. Multiparametric MRI study of ALS stratified for the C9orf72 genotype. Neurology. 2013;81(4):361-9.

58. Bertrand A, Wen J, Rinaldi D, Houot M, Sayah S, Camuzat A, et al. Early Cognitive, Structural, and Microstructural Changes in Presymptomatic C9orf72 Carriers Younger Than 40 Years. JAMA Neurology. 2018;75(2):236-45.

59. Daianu M, Mendez MF, Baboyan VG, Jin Y, Melrose RJ, Jimenez EE, et al. An advanced white matter tract analysis in frontotemporal dementia and early-onset Alzheimer’s disease. Brain Imaging and Behavior. 2016;10(4):1038-53.

60. Downey LE, Mahoney CJ, Buckley AH, Golden HL, Henley SM, Schmitz N, et al. White matter tract signatures of impaired social cognition in frontotemporal lobar degeneration. Neuroimage Clin. 2015;8:640-51.

61. Feis RA, Bouts MJRJ, Dopper EGP, Filippini N, Heise V, Trachtenberg AJ, et al. Multimodal MRI of grey matter, white matter, and functional connectivity in cognitively healthy mutation carriers at risk for frontotemporal dementia and Alzheimer's disease. BMC neurology. 2019;19(1):343-.

62. Floeter MK, Bageac D, Danielian LE, Braun LE, Traynor BJ, Kwan JY. Longitudinal imaging in C9orf72 mutation carriers: Relationship to phenotype. Neuroimage Clin. 2016;12:1035-43.

63. Jakabek D, Power BD, Macfarlane MD, Walterfang M, Velakoulis D, van Westen D, et al. Regional structural hypo- and hyperconnectivity of frontal-striatal and frontal-thalamic pathways in behavioral variant frontotemporal dementia. Hum Brain Mapp. 2018;39(10):4083-93.

64. Mahoney CJ, Beck J, Rohrer JD, Lashley T, Mok K, Shakespeare T, et al. Frontotemporal dementia with the C9ORF72 hexanucleotide repeat expansion: clinical, neuroanatomical and neuropathological features. Brain : a journal of neurology. 2012;135(Pt 3):736-50.

65. Masuda M, Senda J, Watanabe H, Epifanio B, Tanaka Y, Imai K, et al. Involvement of the caudate nucleus head and its networks in sporadic amyotrophic lateral sclerosis-frontotemporal dementia continuum. Amyotroph Lateral Scler Frontotemporal Degener. 2016;17(7-8):571-9.

66. Möller C, Hafkemeijer A, Pijnenburg YAL, Rombouts SARB, van der Grond J, Dopper E, et al. Joint assessment of white matter integrity, cortical and subcortical atrophy to distinguish AD from behavioral variant FTD: A two-center study. Neuroimage Clin. 2015;9:418-29.

67. Panman JL, Jiskoot LC, Bouts MJRJ, Meeter LHH, van der Ende EL, Poos JM, et al. Gray and white matter changes in presymptomatic genetic frontotemporal dementia: a longitudinal MRI study. Neurobiology of Aging. 2019;76:115-24.

68. Papma JM, Jiskoot LC, Panman JL, Dopper EG, den Heijer T, Donker Kaat L, et al. Cognition and gray and white matter characteristics of presymptomatic &lt;em&gt;C9orf72&lt;/em&gt; repeat expansion. Neurology. 2017;89(12):1256.

69. Spotorno N, Lindberg O, Nilsson C, Landqvist Waldö M, van Westen D, Nilsson K, et al. Plasma neurofilament light protein correlates with diffusion tensor imaging metrics in frontotemporal dementia. PLoS One. 2020;15(10):e0236384.

70. Agosta F, Ferraro PM, Riva N, Spinelli EG, Domi T, Carrera P, et al. Structural and functional brain signatures of C9orf72 in motor neuron disease. Neurobiology of Aging. 2017;57:206-19.

71. Dopper EGP, Chalos V, Ghariq E, den Heijer T, Hafkemeijer A, Jiskoot LC, et al. Cerebral blood flow in presymptomatic MAPT and GRN mutation carriers: A longitudinal arterial spin labeling study. Neuroimage Clin. 2016;12:460-5.

72. Farb NA, Grady CL, Strother S, Tang-Wai DF, Masellis M, Black S, et al. Abnormal network connectivity in frontotemporal dementia: evidence for prefrontal isolation. Cortex. 2013;49(7):1856-73.

73. Lee SE, Khazenzon AM, Trujillo AJ, Guo CC, Yokoyama JS, Sha SJ, et al. Altered network connectivity in frontotemporal dementia with C9orf72 hexanucleotide repeat expansion. Brain : a journal of neurology. 2014;137(Pt 11):3047-60.

74. Lee SE, Sias AC, Mandelli ML, Brown JA, Brown AB, Khazenzon AM, et al. Network degeneration and dysfunction in presymptomatic C9ORF72 expansion carriers. Neuroimage Clin. 2016;14:286-97.

75. Lee SE, Sias AC, Kosik EL, Flagan TM, Deng J, Chu SA, et al. Thalamo-cortical network hyperconnectivity in preclinical progranulin mutation carriers. Neuroimage Clin. 2019;22:101751.

76. Ng ASL, Wang J, Ng KK, Chong JSX, Qian X, Lim JKW, et al. Distinct network topology in Alzheimer's disease and behavioral variant frontotemporal dementia. Alzheimer's research & therapy. 2021;13(1):13-.

77. Rijpma MG, Yang WFZ, Toller G, Battistella G, Sokolov AA, Sturm VE, et al. Influence of periaqueductal gray on other salience network nodes predicts social sensitivity. Hum Brain Mapp. 2022;43(5):1694-709.

78. Rombouts SA, van Swieten JC, Pijnenburg YA, Goekoop R, Barkhof F, Scheltens P. Loss of frontal fMRI activation in early frontotemporal dementia compared to early AD. Neurology. 2003;60(12):1904-8.

79. Shoukry RS, Waugh R, Bartlett D, Raitcheva D, Floeter MK. Longitudinal changes in resting state networks in early presymptomatic carriers of C9orf72 expansions. NeuroImage: Clinical. 2020;28:102354.

80. Toller G, Brown J, Sollberger M, Shdo SM, Bouvet L, Sukhanov P, et al. Individual differences in socioemotional sensitivity are an index of salience network function. Cortex. 2018;103:211-23.

81. Zhou J, Greicius MD, Gennatas ED, Growdon ME, Jang JY, Rabinovici GD, et al. Divergent network connectivity changes in behavioural variant frontotemporal dementia and Alzheimer's disease. Brain : a journal of neurology. 2010;133(Pt 5):1352-67.

82. Cistaro A, Pagani M, Montuschi A, Calvo A, Moglia C, Canosa A, et al. The metabolic signature of C9ORF72-related ALS: FDG PET comparison with nonmutated patients. Eur J Nucl Med Mol Imaging. 2014;41(5):844-52.

83. De Vocht J, Blommaert J, Devrome M, Radwan A, Van Weehaeghe D, De Schaepdryver M, et al. Use of Multimodal Imaging and Clinical Biomarkers in Presymptomatic Carriers of C9orf72 Repeat Expansion. JAMA Neurology. 2020;77(8):1008-17.

84. Diehl-Schmid J, Grimmer T, Drzezga A, Bornschein S, Riemenschneider M, Förstl H, et al. Decline of cerebral glucose metabolism in frontotemporal dementia: a longitudinal 18F-FDG-PET-study. Neurobiol Aging. 2007;28(1):42-50.

85. Diehl-Schmid J, Licata A, Goldhardt O, Förstl H, Yakushew I, Otto M, et al. FDG-PET underscores the key role of the thalamus in frontotemporal lobar degeneration caused by C9ORF72 mutations. Translational Psychiatry. 2019;9(1):54.

86. Frisch S, Dukart J, Vogt B, Horstmann A, Becker G, Villringer A, et al. Dissociating memory networks in early Alzheimer's disease and frontotemporal lobar degeneration - a combined study of hypometabolism and atrophy. PLoS One. 2013;8(2):e55251.

87. Grimmer T, Diehl J, Drzezga A, Förstl H, Kurz A. Region-specific decline of cerebral glucose metabolism in patients with frontotemporal dementia: a prospective 18F-FDG-PET study. Dement Geriatr Cogn Disord. 2004;18(1):32-6.

88. Ishii K, Sakamoto S, Sasaki M, Kitagaki H, Yamaji S, Hashimoto M, et al. Cerebral glucose metabolism in patients with frontotemporal dementia. J Nucl Med. 1998;39(11):1875-8.

89. Jang YK, Lyoo CH, Park S, Oh SJ, Cho H, Oh M, et al. Head to head comparison of [(18)F] AV-1451 and [(18)F] THK5351 for tau imaging in Alzheimer's disease and frontotemporal dementia. Eur J Nucl Med Mol Imaging. 2018;45(3):432-42.

90. Jeong Y, Cho SS, Park JM, Kang SJ, Lee JS, Kang E, et al. 18F-FDG PET findings in frontotemporal dementia: an SPM analysis of 29 patients. J Nucl Med. 2005;46(2):233-9.

91. Leuzy A, Zimmer ER, Dubois J, Pruessner J, Cooperman C, Soucy JP, et al. In vivo characterization of metabotropic glutamate receptor type 5 abnormalities in behavioral variant FTD. Brain Struct Funct. 2016;221(3):1387-402.

92. Malpetti M, Holland N, Jones PS, Ye R, Cope TE, Fryer TD, et al. Synaptic density in carriers of C9orf72 mutations: a [(11) C]UCB-J PET study. Ann Clin Transl Neurol. 2021;8(7):1515-23.

93. Matias-Guiu JA, Cabrera-Martín MN, Fernádez-Matarrubia M, Moreno-Ramos T, Valles-Salgado M, Porta-Etessam J, et al. Topography of primitive reflexes in dementia: an F-18 fluorodeoxyglucose positron emission tomography study. Eur J Neurol. 2015;22(8):1201-7.

94. Poljansky S, Ibach B, Hirschberger B, Männer P, Klünemann H, Hajak G, et al. A visual [18F]FDG-PET rating scale for the differential diagnosis of frontotemporal lobar degeneration. Eur Arch Psychiatry Clin Neurosci. 2011;261(6):433-46.

95. Popuri K, Beg MF, Lee H, Balachandar R, Wang L, Sossi V, et al. FDG-PET in presymptomatic C9orf72 mutation carriers. NeuroImage: Clinical. 2021;31:102687.

96. Schaeverbeke J, Gabel S, Meersmans K, Bruffaerts R, Liuzzi AG, Evenepoel C, et al. Single-word comprehension deficits in the nonfluent variant of primary progressive aphasia. Alzheimers Res Ther. 2018;10(1):68.

97. Soleimani-Meigooni DN, Iaccarino L, La Joie R, Baker S, Bourakova V, Boxer AL, et al. 18F-flortaucipir PET to autopsy comparisons in Alzheimer's disease and other neurodegenerative diseases. Brain. 2020;143(11):3477-94.
